# Restoring cytonuclear harmony: Distinct strategies in Arabidopsis auto‐ and allopolyploids

**DOI:** 10.1111/tpj.70451

**Published:** 2025-08-28

**Authors:** Mehrdad Shahbazi, Jana Kneřová, Denisa Kubíková, Alžběta Doležalová, Marek Szecówka, Yasmim Dutra Santos, Jonathan F. Wendel, Joel Sharbrough, David Kopecký

**Affiliations:** ^1^ Centre of Plant Structural and Functional Genomics Institute of Experimental Botany of the Czech Academy of Sciences Olomouc Czech Republic; ^2^ National Centre for Biomolecular Research, Faculty of Science Masaryk University Brno Czech Republic; ^3^ Department of Ecology, Evolution, and Organismal Biology Iowa State University Ames Iowa USA; ^4^ Department of Ecology, Evolution, and Marine Biology University of California Santa Barbara California USA

**Keywords:** mitochondria, chloroplasts, cytonuclear interactions, gene expression, organellar DNA, interspecific hybridization, whole genome duplication, stoichiometry

## Abstract

Plants rely on tight coordination between nuclear, mitochondrial, and chloroplast genomes to form essential multi‐enzyme cytonuclear complexes. Whole‐genome duplication (WGD) doubles the nuclear genome, potentially disrupting cytonuclear stoichiometry unless organellar genomes respond accordingly. Targeted analyses of chloroplasts and mitochondria enabled us to dissect the extent and mechanisms of adjustments in both organelles immediately after WGD and across generations in Arabidopsis auto‐ and allopolyploids. We observed a substantial overcompensation of organellar genome copies in both organelles in early‐generation autotetraploids primarily through multiplication of DNA copies within organelles rather than increasing the number of organelles. Despite higher DNA content, mitochondria maintained their volume, and chloroplasts were even smaller. In successive generations, chloroplast DNA copy numbers continued to rise, whereas mitochondrial DNA copies declined. Gene expression patterns also differed between chloroplasts and mitochondria and between auto‐ and allopolyploids. In autopolyploids, immediate transcriptional changes were minimal, but by the fourth generation after WGD, nuclear genes involved in mitochondria‐nuclear complexes were downregulated. In allopolyploids, transcriptional changes appeared immediately in the first generation (chloroplast genes were upregulated and mitochondrial genes were downregulated). Our findings demonstrate that cytonuclear balance is restored through dynamic, organelle‐specific, and polyploid‐type‐specific mechanisms. These insights advance our understanding of the evolution of polyploid genomes.

## Significance Statement

Whole‐genome duplication (WGD) disrupts cytonuclear balance by doubling nuclear content, requiring compensatory responses from organelles. In Arabidopsis auto‐ and allopolyploids, we found dynamic, organelle‐specific mechanisms (primarily increased organellar DNA copy number rather than organelle proliferation) to restore stoichiometry. Mitochondria and chloroplasts adjusted differently across generations, with distinct gene expression changes depending on polyploid type. These findings reveal that cytonuclear coordination after WGD involves complex, evolving responses, advancing our understanding of genome evolution in polyploid plants.

## INTRODUCTION

In plants, DNA is stored in three distinct compartments: the nucleus, mitochondria, and chloroplasts. The latter two organelles evolved via endosymbiosis of ancestral bacteria and still retain part of their genomes, including up to 70 (mitochondria) and 120–130 (chloroplast) genes (Butenko et al., 2024; Daniell et al., 2016). Shortly after these endosymbiotic events occurred, most organellar genes were transferred to nuclear genomes. As a result, protein complexes involved in vital biological processes, such as photosynthesis and oxidative phosphorylation, entail intimate physical interactions between organelle‐ and nucleus‐encoded genes (Camus et al., 2022; Rand et al., 2004; Sloan et al., 2018). A textbook example is the protein complex RuBisCo, a heterohexadecamer comprised of the chloroplast‐encoded large subunit (*LSU*) and the nuclear‐encoded small subunit (*SSU*), the latter of which must be imported into the chloroplast from the cytoplasm (Tabita et al., 2007). Expression of nuclear and organellar genes involved in cytonuclear complexes must therefore be coordinated so that plants can successfully assimilate and metabolize carbon. This essential stoichiometry may be disturbed by a change in the ratio of nuclear to organellar gene copy numbers, which can occur from duplication or deletion of individual genes or as a consequence of whole genome duplication (WGD; Sémon & Wolfe, 2007).

Although natural polyploids may be derived in a variety of ways, they are classically divided between autopolyploids, which have multiple copies of nearly identical genomes, and allopolyploids, formed from hybridization and thus containing duplicated (homoeologous) sets of variously divergent chromosomes (Barker et al., 2016; Stebbins, 1947). Although polyploidization is an important mechanism of plant evolution and speciation (Heslop‐Harrison et al., 2023; Soltis et al., 2009), newly emerged polyploids face challenges associated with pairing and segregation of multiple copies of chromosomes during meiotic division (Gonzalo et al., 2023), altered transcriptional regulation and epigenetic reprogramming (Comai, 2005; Teng et al., 2023), and disturbance to cytonuclear interactions (Shahbazi et al., 2024; Sharbrough et al., 2017). Responses to disrupted stoichiometry include an increase in the number of organelles and/or organellar genome number, downregulation of nuclear gene expression and/or upregulation of organellar gene expression for cytonuclear complexes (Fernandes Gyorfy et al., 2021; Sloan et al., 2024). In addition, the immediate responses to WGD in the first generation may be different from those of successive generations (Shahbazi et al., 2024).

In general, polyploids possess a higher number of organelles than their diploid counterparts, even though the extent of this increase varies among different species, from about 1.7‐fold increase in chloroplast numbers in sugar beet to about 1.15‐fold increase in *Festuca pratensis* compared to their diploid counterparts (Shahbazi et al., 2024). In *F. pratensis*, the initial increase is adjusted in further generations; newly established autopolyploids (C_0_ generation) have only a 1.15‐fold increase in contrast to a 1.35‐fold increase observed in the advanced (C_8_) generation (Shahbazi et al., 2024). These observations imply that an increase in the number of chloroplasts only partially compensates for the imbalance in the cytonuclear stoichiometry following WGD and suggest that other mechanisms also are involved. Less is understood about mitochondrial numbers (Doyle & Coate, 2019), perhaps because of difficulties in quantifying mitochondria, which undergo frequent fusions and fissions in response to the cellular environment (Arimura et al., 2004; Logan, 2006; Sheahan et al., 2005; van der Zutphen & Klei, 2011).

In addition to modifications in organelle number, stoichiometry restoration in polyploid flowering plants commonly entails adjustments in organellar genome copy number per organelle (Doyle & Coate, 2019; Fernandes Gyorfy et al., 2021; Lim et al., 2020; Ma & Li, 2015). Relatively few studies, however, have simultaneously investigated both organelle numbers and organellar genome copy numbers. Consequently, it is often difficult to determine the individual contributions of each mechanism. An example of this approach is for the newly created autotetraploid *F. pratensis* and *Lolium multiflorum*, where Shahbazi et al. (2024) found that there were only 1.35× and 1.2× more chloroplasts, respectively, but 2.5× more chloroplast genome (cpDNA) copies compared to their diploid counterparts. Such overcompensation is not exceptional, as it also has been observed in tetraploid wheat (*Triticum turgidum*; Fernandes Gyorfy et al., 2021). One might expect that overcompensation would be adjusted in further generations to reach a level approximately that of doubled nuclear genes, all else being equal. In contrast, Shahbazi et al. (2024) observed even more pronounced increases in the copy numbers of cpDNA in advanced (C_7_–C_8_) generations of autotetraploid *F. pratensis*, relative to the first generation after WGD.

Several intriguing aspects of cytonuclear interactions remain to be explored. Are stoichiometric responses to autopolyploidy different from those of allopolyploidy? What is the timing and dynamics of stoichiometry adjustments? Is it an immediate consequence of polyploidization in plants, as suggested by Sloan et al. (2024), or do changes accrue gradually in the generations following initial WGD, or some combination of these two temporal dimensions? Are the mechanisms underlying stoichiometric evolution in plant polyploids similar for mitochondria and chloroplasts?

The aim of this paper is to address the timing and nature of stoichiometric adjustment to WGD using two types of polyploids, auto‐ and allo‐, using newly established polyploids of the same origin (*Arabidopsis thaliana*, *Arabidopsis lyrata* and their diploid and allopolyploid hybrids). We investigated the dynamics and adjustments of cytonuclear stoichiometry across successive generations, including changes in the number of organellar genome copies, organelle (chloroplast and mitochondria) numbers and volumes, and gene expression patterns. Our findings suggest that WGD affects cytonuclear interactions differently in auto‐ and allopolyploids, with mitochondria and chloroplasts exhibiting different levels and trajectories of responses.

## RESULTS

For analyses, we used sets of diploid and tetraploid *A. thaliana* and *A. lyrata*, and their interspecific hybrids (*A. thaliana* ♀ × *A. lyrata* ♂). Tetraploids were produced by colchicine treatment of diploids to provide direct comparisons of diploids versus tetraploids having the same genetic background. Ploidy levels of all plants were confirmed by flow cytometry and five confirmed tetraploids (C_0_) were selfed to produce C_1_ seed. In the case of *A. thaliana*, we produced the next three generations of autotetraploids, and plants of the C_4_ generation were also used in our analyses (Figure S1). C_0_ plants of allotetraploid *A. thaliana* × *A. lyrata* were mostly sterile, and analyses were done at C_0_ generation.

### Autopolyploids exhibit a more robust compensatory increase in the number of chloroplasts than allopolyploids

**Figure 1:**
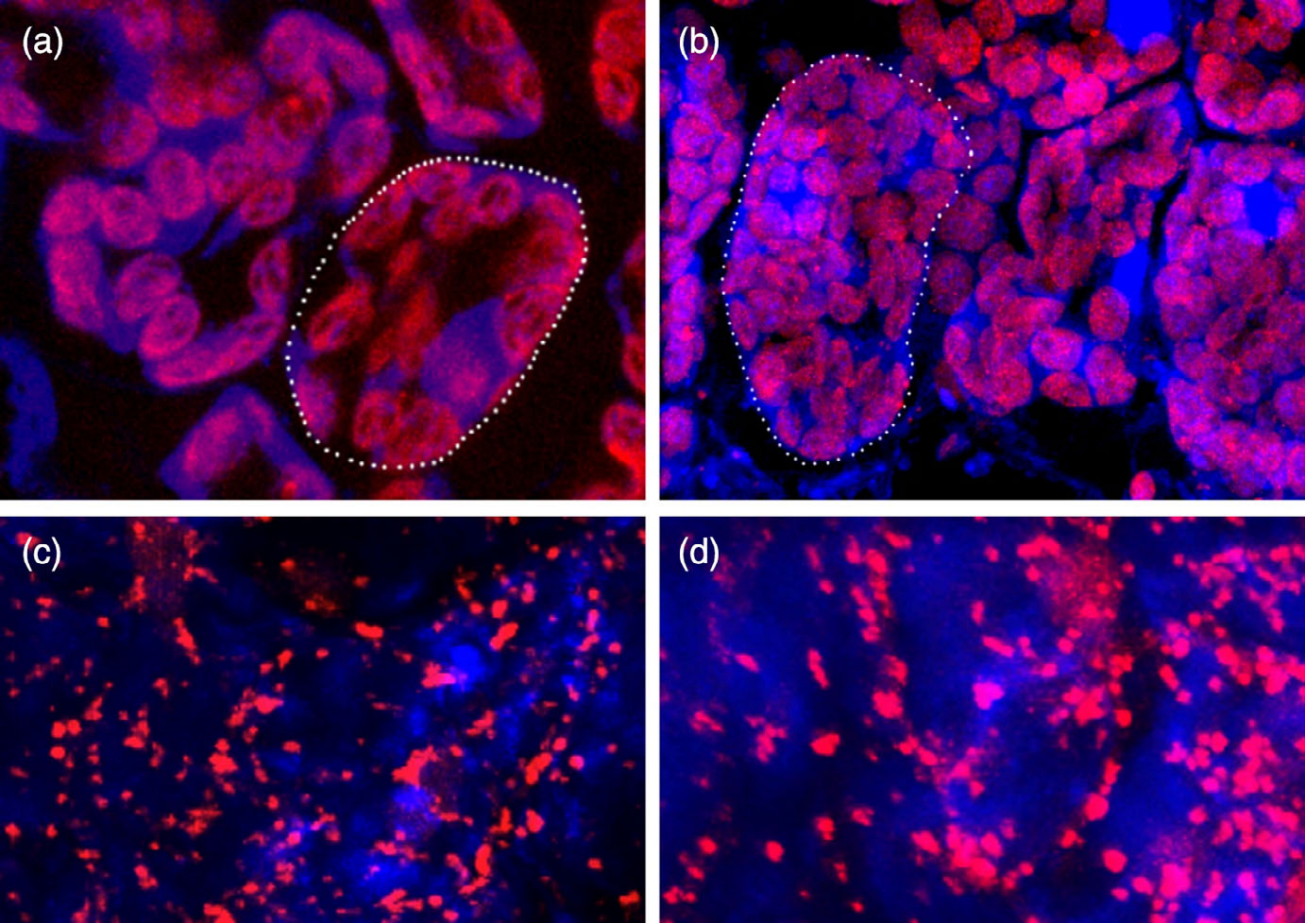
Visualization of chloroplasts and mitochondria in *Arabidopsis thaliana*. (a, b) Chloroplasts were visualized using autofluorescence in cells of diploid (a) and autotetraploid (b) genotypes of *A. thaliana*. (c, d) Individual cells are highlighted by dotted lines. Mitochondria were visualized using the CFP marker line in diploids (c) and autotetraploid (d) *A. thaliana*.

We first investigated changes in cell morphology after WGD, focusing on both chloroplasts and mitochondria (Figure 1). Specifically, we compared the number and volume of chloroplasts between diploids and their corresponding auto‐ and allotetraploids, with three to five plants per population examined. Analyzing volumes and numbers of mitochondria, however, is technically more demanding than chloroplasts due to the need for staining, their high number per cell, and their frequent fusion and fission. Despite these difficulties, we succeeded in collecting data on the number and volume of mitochondria in diploid and autotetraploid *A. thaliana*. We utilized a publicly available *A. thaliana* mitochondria cyan fluorescent protein (CFP) marker line mt‐ck (Nelson et al., 2007). To make the analysis more manageable, we estimated the number and total volume of mitochondria per constant size of the Z‐stack image generated by the confocal microscope (185 × 185 × 15 μm) rather than per cell.

**Figure 2:**
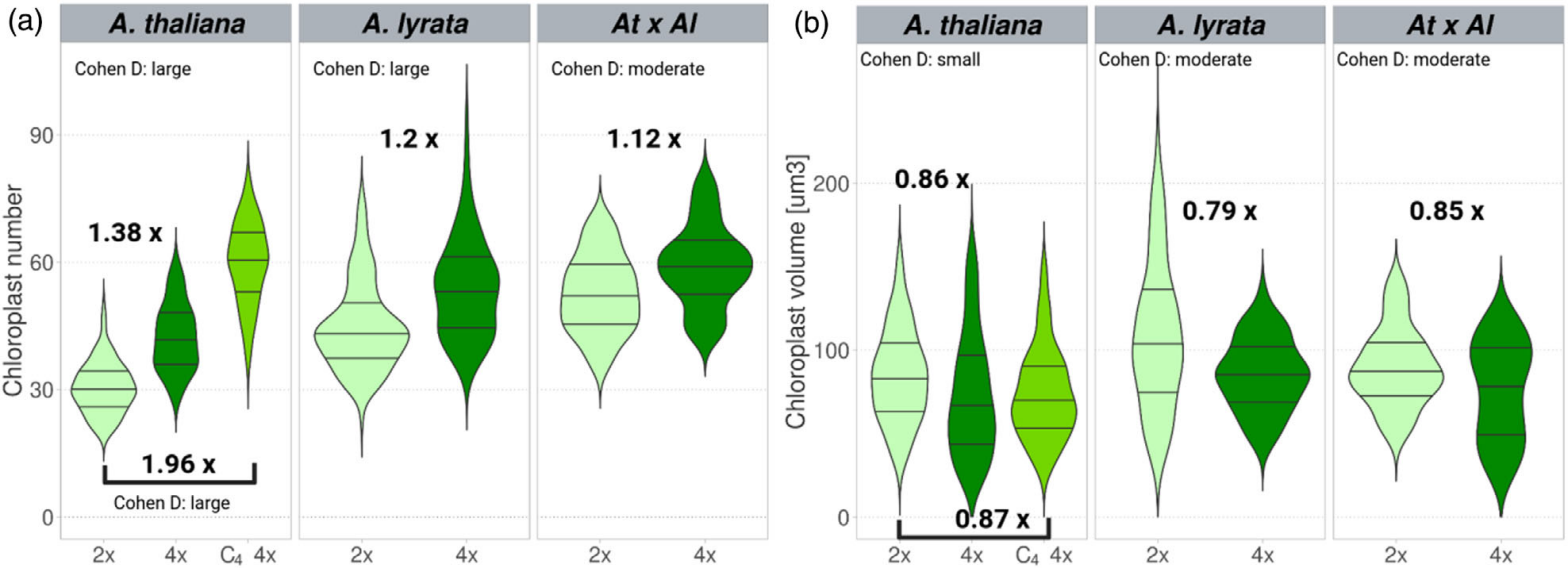
Changes in chloroplast number and size after WGD in *Arabidopsis thaliana*, *Arabidopsis lyrata*, and *A. thaliana* × *A. lyrata* hybrids. (a, b) The *y* axis represents the number of chloroplasts (a) and chloroplast volume (b) per cell. Mean differences between diploids (light green) and tetraploids (C_1_ in dark green and C_4_ in olive green) are expressed as fold change in the tetraploids. The effect size of the difference between the two means is represented by Cohen's *d* test. Negligible effect *d* < 0.2; small effect *d* =0.2–0.5; moderate effect *d* =0.5–0.8; large effect *d* ≥ 0.8; *n*, number of cells evaluated.

**Figure 3:**
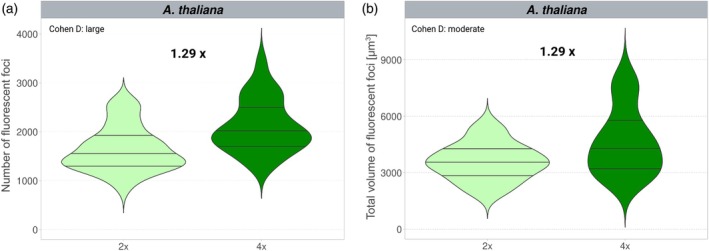
Changes in mitochondria number and volume after WGD in *Arabidopsis thaliana*. (a, b) *y* axis represents the number of fluorescent foci (a) and total volume of fluorescent foci (b) per Z‐stack volume in *A. thaliana* CFP labeled mitochondria marker line. Mean differences between diploids (light green) and tetraploids (dark green) are expressed as fold change in the tetraploids. The effect size of the difference between the two means is represented by Cohen's *d* test. Negligible effect *d* < 0.2; small effect *d* =0.2–0.5; moderate effect *d* =0.5–0.8; large effect *d* ≥ 0.8; *n*, number of cells evaluated.

We observed a 1.38‐fold and 1.20‐fold (*P* = 0.003 and *P* < 0.001) increase in the number of chloroplasts in autotetraploids compared to diploids in *A. thaliana* and *A. lyrata*, respectively (Figure 2a). Specifically, autopolyploid *A. thaliana* contained 42.3 ± 0.7 (mean ± SE) chloroplasts per cell versus 30.4 ± 0.5 in diploids, and autopolyploid *A. lyrata* contained 54 ± 1 chloroplasts per cell versus 45 ± 1 in diploids. This represented a large effect in both species (*A. thaliana*: *d* = 1.7; *A. lyrata*: *d* = 1.2). In contrast, the increase was less pronounced in the *A. thaliana* × *A. lyrata* allopolyploids, with only a 1.12‐fold increase in chloroplast number over diploid hybrids (59 ± 1 vs. 53 ± 1; *P* < 0.001, *d* = 0.4; Figure 2a). Together, these data indicate that while all *Arabidopsis* polyploids harbor greater numbers of chloroplasts per cell compared to diploids, autopolyploids appear to respond more robustly in this regard than allopolyploids to the change in nuclear genome copy number. The number of mitochondria, represented by the number of CFP foci, increased in the autotetraploid mt‐ck *A. thaliana* line compared to diploid progenitors by 1.29‐fold (*P* < 0.001, *d* = 0.9) representing a large effect (Figure 3a; Data S1). The increase in the number of organelles in polyploids is therefore very similar in mitochondria and chloroplasts.

To explore the possibility of change over generations, we also examined the C_4_ generation of autotetraploid *A. thaliana*. While the increase in the number of chloroplasts in the C_1_ generation was 1.38‐fold (see above), the C_4_ generation harbored 1.96‐fold (*P* < 0.001, *d* = 3.6) as many chloroplasts (59.8 ± 0.8) compared to diploids (30.4 ± 0.5). The implications are that the immediate increase in the number of chloroplast numbers following WGD appears to only partially compensate for the stoichiometric imbalance produced by the increased number of genomes, with additional compensatory increases in the number of chloroplasts occurring in the succeeding generations.

### Chloroplast volumes are reduced in polyploids compared to diploids

Because organelle size offers an additional mechanism whereby polyploids could respond to post‐WGD life in which their doubled nuclear genomes accommodate larger cells, we hypothesized that polyploids might harbor larger chloroplasts and mitochondria than diploids, in addition to the increased number. We employed a Z‐stack approach using confocal microscopy to quantify total chloroplast (on a per cell basis) and mitochondria (on a per constant Z‐stack volume basis—see Experimental Procedures) volumes in leaf tissue and compared them across diploid and tetraploid plants, as before. In all cases (*A. thaliana*, *A. lyrata* and *A. thaliana* × *A. lyrata*), chloroplast volumes were similarly reduced in tetraploids compared to diploids, with a 0.86‐fold (*P* = 0.002, *d* = 0.3), 0.79‐fold (*P <* 0.001, *d* = 0.4) and 0.85‐fold (*P* = 0.001, *d* = 0.4) reduction, respectively (Figure 2b). However, the effect size of this difference is in all cases small. In mitochondria, total volume per constant Z‐stack size increased in autotetraploids (1.29‐fold increase; *P <* 0.001, *d* = 0.9), but this was proportional to the increase of mitochondria number (also 1.29‐fold increase; *P <* 0.001, *d* = 0.7), suggesting no change in the volume of mitochondria (Figure 3b; Data S1).

Specifically, we found that the average chloroplast volume was 85 ± 2 μm^3^ and 73 ± 3 μm^3^ in diploid and tetraploid *A. thaliana*, 108 ± 4 and 86 ± 2 μm^3^ in diploid and tetraploid *A. lyrata*, and 90 ± 2 and 76 ± 4 μm^3^ in diploids and tetraploids of *A. thaliana* × *A. lyrata*, respectively. Similar reduction was also observed in the C_4_ generation of autotetraploid *A. thaliana*, where the chloroplast volume was 74 ± 2 μm^3^, which was 0.87‐fold of the volume of diploid progenitors (85 ± 2 μm^3^; Figure 2b). These results indicate firstly (and surprisingly) that polyploids do not compensate for altered cytonuclear stoichiometry by increasing the volume of their chloroplasts and mitochondria. Secondly, and more broadly, the joint observations of increased chloroplast number but decreased chloroplast volume in polyploids compared to diploids are consistent with a scenario in which chloroplast surface area is a more important constraint on photosynthetic performance than chloroplast volume.

### Overcompensation in copy number of organellar genomes in polyploids

Previous work has shown that both natural and synthetic polyploid plants exhibit elevated organelle genome copy number compared to diploid relatives (Fernandes Gyorfy et al., 2021; Shahbazi et al., 2024). To test whether this was different in auto‐ versus allopolyploids and whether the degree of organelle genome copy number compensation changed across generations, we estimated copy number for four chloroplast genes (ATP synthase subunit I*—atpI*; photosystem II D2 protein—*psbD*; cytochrome f apoprotein—*petA*; and NAD(P)H dehydrogenase subunit B—*ndhB*) and three mitochondrial genes (cytochrome c oxidase subunit I—*cox1*; ribosomal protein S3—*rps3*; ATP synthase membrane subunit 6—*atp6*), all involved in cytonuclear complexes, using droplet digital PCR (ddPCR) in the same diploids and tetraploids described above. All data are reported on a per‐nuclear genome copy basis for diploid and tetraploid samples. The number of organellar gene copies was divided by the number of nuclear (photosystem II subunit R (*PSBR*) for chloroplasts and ATP synthase subunit beta‐1 (*ATPβ1*) for mitochondria) gene copies. We then compared tetraploids to corresponding diploids by taking the tetraploid: diploid ratio. When these across‐ploidy ratios were close to 1, we called this ‘compensation’ (i.e., tetraploids exhibit a similar cytonuclear gene dosage balance as diploids), >1 suggests ‘overcompensation’ and <1 implies ‘undercompensation’. We applied a mixed‐effects modeling approach carried out using RStudio to evaluate whether gene and ploidy were significant predictors of organellar genome copy number. In most cases, genes did not significantly contribute to the variation in genome copy number estimates (Data S3), with the exceptions of cpDNA copy variation in *A. lyrata* (*χ* ^2^ = 10.899, d.f. = 3, *P* = 0.012) and mtDNA copy variation in *A. thaliana* C_4_ (*χ* ^2^ = 3.173, d.f. = 2, *P* = 0.0013).

**Figure 4:**
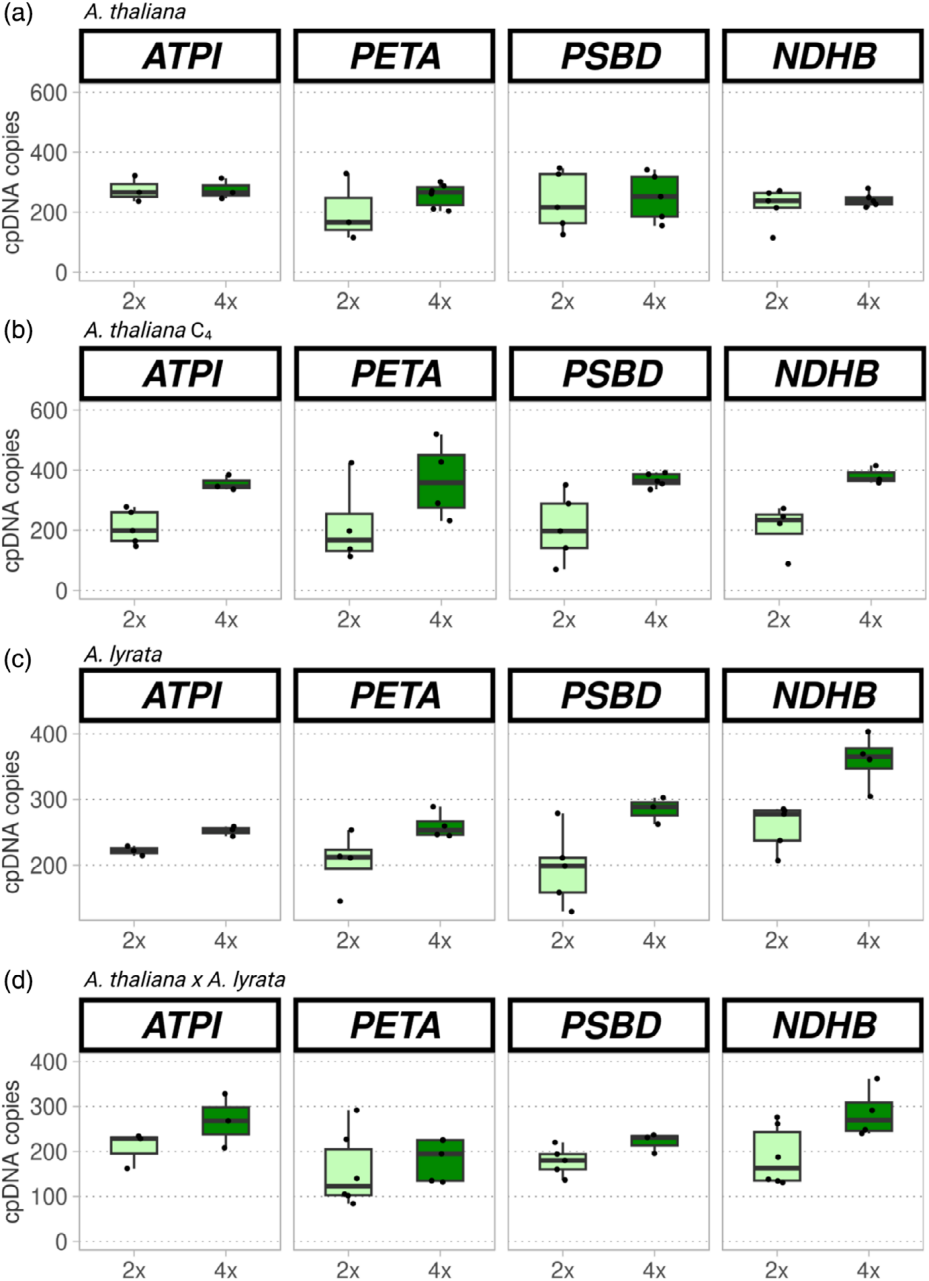
Changes in cpDNA copy number after WGD in leaves of *Arabidopsis thaliana*, *Arabidopsis lyrata*, and *A. thaliana* × *A. lyrata* hybrids. (a–d) Each plot represents the number of cpDNA copies per nuclear genome copy (*y* axis) of diploid (light green) and corresponding tetraploid (dark green) plants based on ddPCR of several chloroplast‐encoded genes in *A. thaliana* C_1_ (a), *A. thaliana* C_4_ (b), *A. lyrata* (c) and *A. thaliana* **×** *A. lyrata* (d). Chloroplast gene NAD(P)H‐quinone oxidoreductase subunit 2 (*NDHB*) was included as a control. Its value was divided by two because it has two copies per cpDNA.

In *A. thaliana*, we found that the increase in cpDNA copies after WGD was from 301 ± 34 in diploids to 317 ± 27 in C_1_ autotetraploids. On average, this translated into a 1.05‐fold change. Based on the model, ploidy was not a significant predictor of genome copy number per nuclear genome copy in this instance, suggesting compensation (*χ* ^2^ = 0.102, d.f. = 1, *P* = 0.749; Figure 4a). By contrast, after three generations, C_4_ tetraploids exhibited a 1.74‐fold increase in the cpDNA copies per nuclear genome copy (447 ± 49 vs. 257 ± 33 in diploids; *χ* ^2^ = 4.961, d.f. = 1, *P* = 0.026; Figure 4b), indicating overcompensation. Diploid *A. lyrata* exhibited a similar number of cpDNA copies per nuclear genome copy (297 ± 36) as *A. thaliana*, and autopolyploids exhibited only a slight, but not significant, 1.33‐fold increase in the cytonuclear gene dosage balance compared to diploids (395 ± 59 cpDNA copies per nuclear genome copy; *χ* ^2^ = 1.023, d.f. = 1, *P* = 0.312; Figure 3c), indicating adequate compensation for genome duplication. Allopolyploids displayed similar degrees of compensation as autopolyploids (1.33‐fold; *χ* ^2^ = 1.623, d.f. = 1, *P* = 0.203; Figure 4d), with diploid hybrids exhibiting 233 ± 30 cpDNA copies per nuclear genome copy and polyploid hybrids exhibiting 310 ± 46 cpDNA copies per nuclear genome copy.

**Figure 5:**
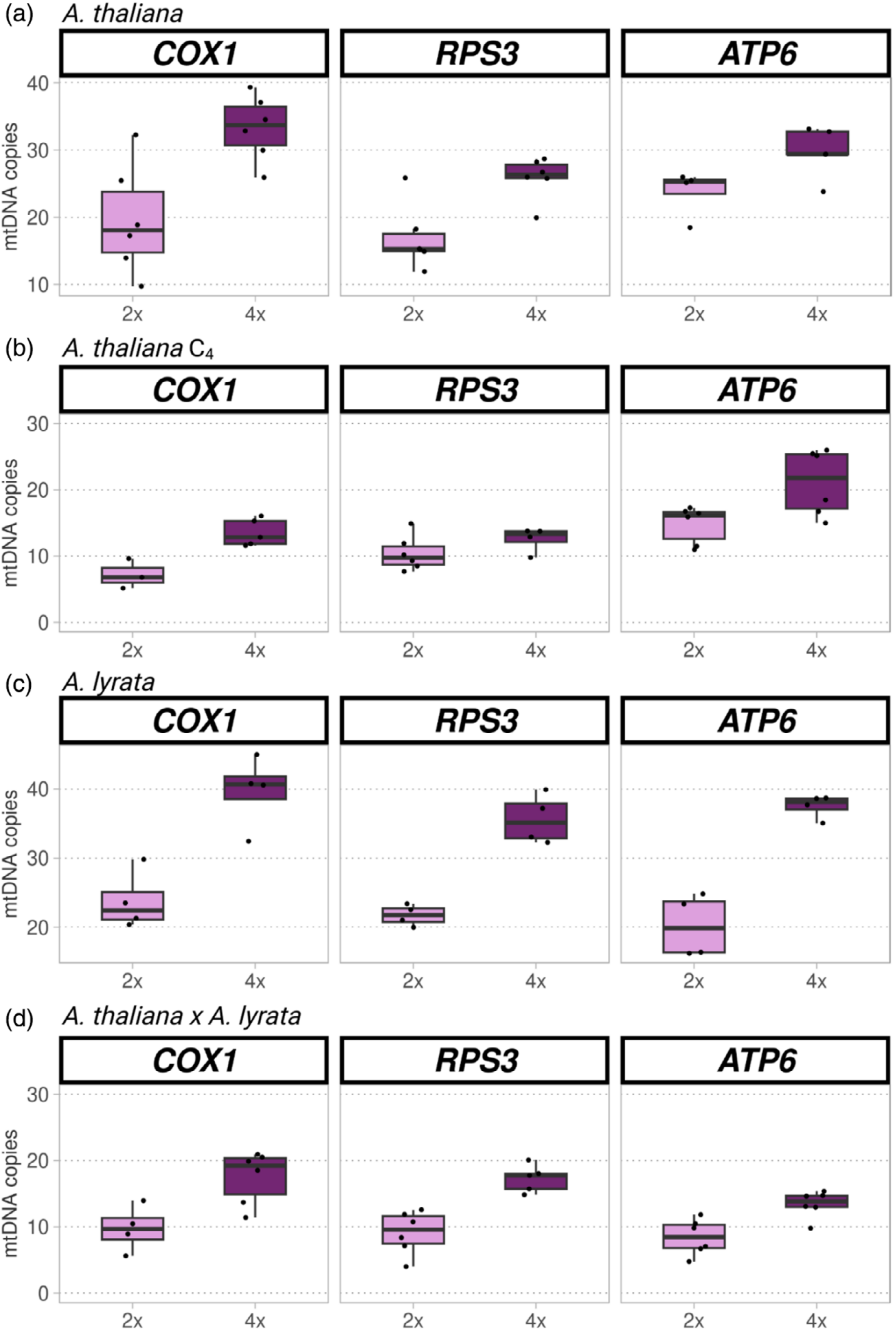
Changes in mtDNA copy number after WGD in leaves of *Arabidopsis thaliana*, *Arabidopsis lyrata*, and *A. thaliana* × *A. lyrata* hybrids. (a–d) Each plot represents the number of mtDNA copies per nuclear genome copy (*y* axis) of diploid (pink) corresponding autotetraploid (purple) plants based on ddPCR of several mitochondrially encoded genes in *A. thaliana* C1 (a), *A. thaliana C4* (b), *A. lyrata* (c) and *A. thaliana* **×** *A. lyrata* (d). Mitochondria gene ATP synthase membrane subunit 6 (*ATP6*) was included as a control. Its value was divided by two because it has two copies per mtDNA.

We observed a slightly different pattern in mtDNA: all newly established polyploids exhibited evidence of overcompensation with substantially higher relative levels of mitochondrial gene copies in tetraploids than in diploids, but this difference appeared to abate across generations. Specifically, we found that *A. thaliana* autotetraploids (25 ± 4 mtDNA copies per nuclear genome copy) exhibited a 1.4‐fold increase in the mitonuclear gene dosage balance compared to diploids (18 ± 3 mtDNA copies per nuclear genome copy; Figure 5a). However, this increase was not statistically significant (*χ* ^2^ = 3.2535, d.f. = 1, *P* = 0.071). *A. lyrata* autotetraploids (50 ± 5 mtDNA copies per nuclear genome copy) exhibited significant overcompensation: a 1.8‐fold increase in the mitonuclear gene dosage balance compared to diploids (29 ± 3 mtDNA copies per nuclear genome copy; *χ* ^2^ = 42.621, d.f. = 1, *P* < 0.001; Figure 5c). Allopolyploid *A. thaliana* × *A. lyrata* (21 ± 1 mtDNA copies per nuclear genome copy) also exhibited significant overcompensation with a 1.7‐fold increase in the mitonuclear gene dosage balance compared to diploids (12 ± 1 mtDNA copies per nuclear genome copy; *χ* ^2^ = 8.299, d.f. = 1, *P* = 0.004; Figure 5d). By contrast, C_4_ autotetraploids (25 ± 4 mtDNA copies per nuclear genome copy) exhibited a 1.4‐fold increase compared to C_4_ diploids (18 ± 3 mtDNA copies per nuclear genome copy; *χ* ^2^ = 12.307, d.f. = 1, *P* < 0.001; Figure 5b).

We conclude that for the autotetraploids and allotetraploids studied here, WGD results in compensation in cpDNA and overcompensation in mtDNA copy number immediately accompanying polyploidization, and that for cpDNA, but not mtDNA, the magnitude of this compensation increases until at least the C_4_ generation.

### Allopolyploids, but not autopolyploids, show significant changes in organellar gene expression

We used quantitative real‐time PCR (qRT‐PCR) to study the expression of genes either involved or not involved in cytonuclear complexes in diploids and tetraploids. For this, we estimated the level of transcription of the chloroplast genes *ATPI*, *PETA*, *PSBD*, and their nuclear counterparts ATP synthase gamma chain 1 (*ATPC1*), photosynthetic electron transfer C (*PETC*) and *PSBR*; and the mitochondrial genes *COX1* and *RPS3* along with their nuclear counterparts, ribosomal protein L33 family protein (*BL33M*) and cytochrome c oxidase 10 (*COX10*). As ontrols, we analyzed the expression of nuclear (organelle‐targeted) and organellar non‐interacting genes, including the chloroplast genes Chloroplast DNA‐dependent RNA polymerase B subunit (*RPOB*) and RNA polymerase beta' subunit‐2 (*RPOC2*); the nuclear chloroplast‐targeted genes *Arabidopsis* Twinkle Homolog (*ATH*), PsbP domain‐containing protein 7 (*PPD7*), and Polymerase gamma 2 (*POLGAMMA2*); the mitochondrial genes Uncharacterized tatC‐like protein ymf16 (*MTT2*) and Cytochrome c biogenesis orf203 (*ORF203*); and the nuclear‐encoded, mitochondria‐targeted genes mitochondrial malate dehydrogenase 1 (*mMDH1*), Glutaredoxin S15 (*GRXS15*), and Dicarboxylate carrier 3 (*DIC3*). As controls, we used genes that are not involved in cytonuclear complexes, that is, the nuclear genes ACTIN 2 (*ACT2*) and eukaryotic initiation factor 4A (*EIF4A*), chloroplast Maturase K (*MATK*) and mitochondrial Intron maturase (*MATR*). All data are reported as relative expression levels in diploid and tetraploid samples.

**Figure 6:**
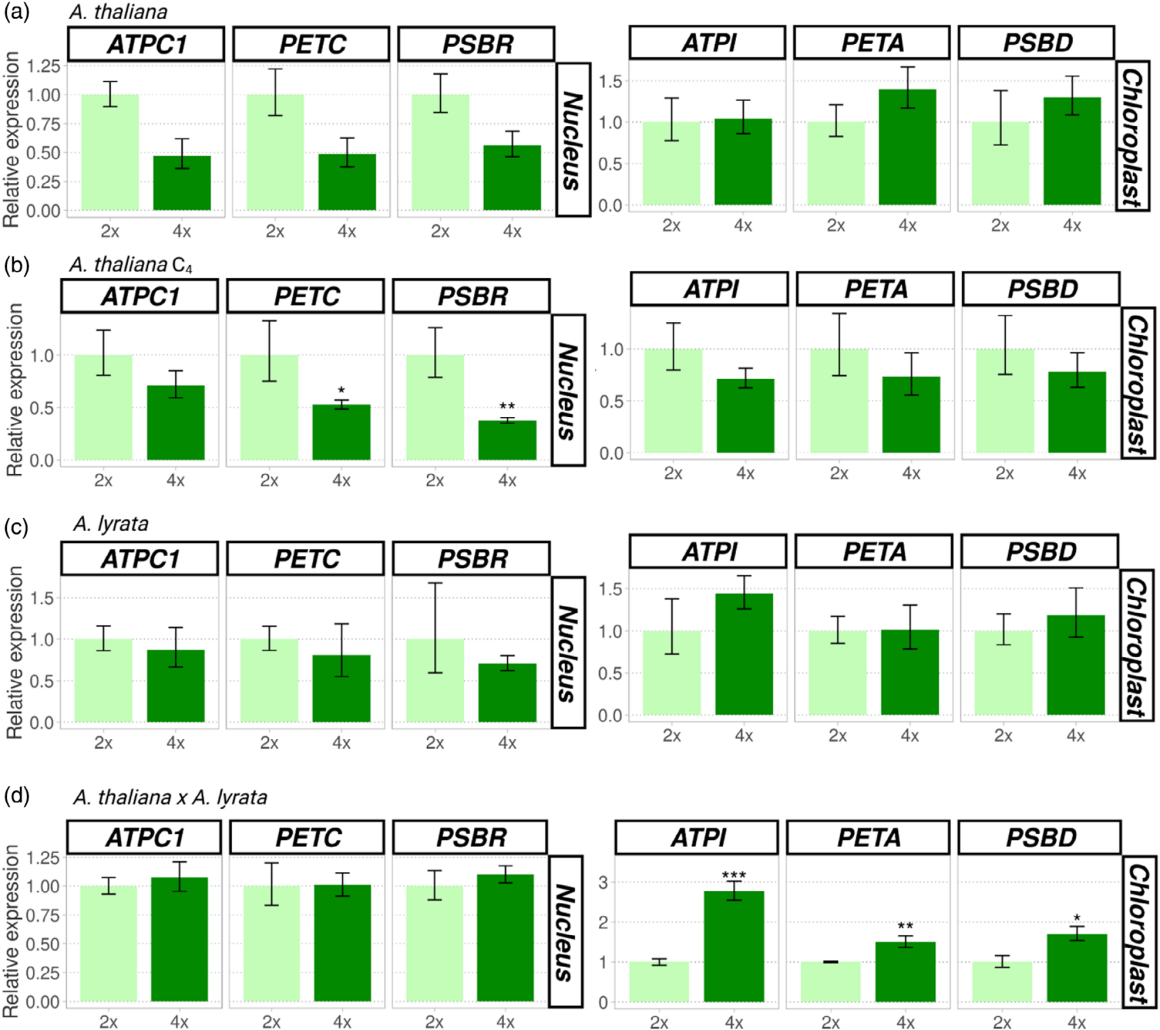
Changes in expression of cytonuclear genes involved in chloroplast complexes after WGD in leaves of *Arabidopsis thaliana*, *Arabidopsis lyrata*, and *A. thaliana × A. lyrata* hybrids. (a–d) Each plot represents the relative transcript abundance (*y* axis) of nuclear‐encoded (left) and chloroplast‐encoded genes (right) involved in cytonuclear complexes in diploid (light green) and corresponding polyploid (dark green) plants based on qRT‐PCR in *A. thaliana* C1 (a), *A. thaliana* C4 (b), *A. lyrata* (c) and *A. thaliana* × *A. lyrata* (d). Error bars represent the standard error of the mean of the biological replicates. ***P < 0.001, **P < 0.01, *P < 0.05 (based on two‐tailed Student's *t*‐test).

**Figure 7:**
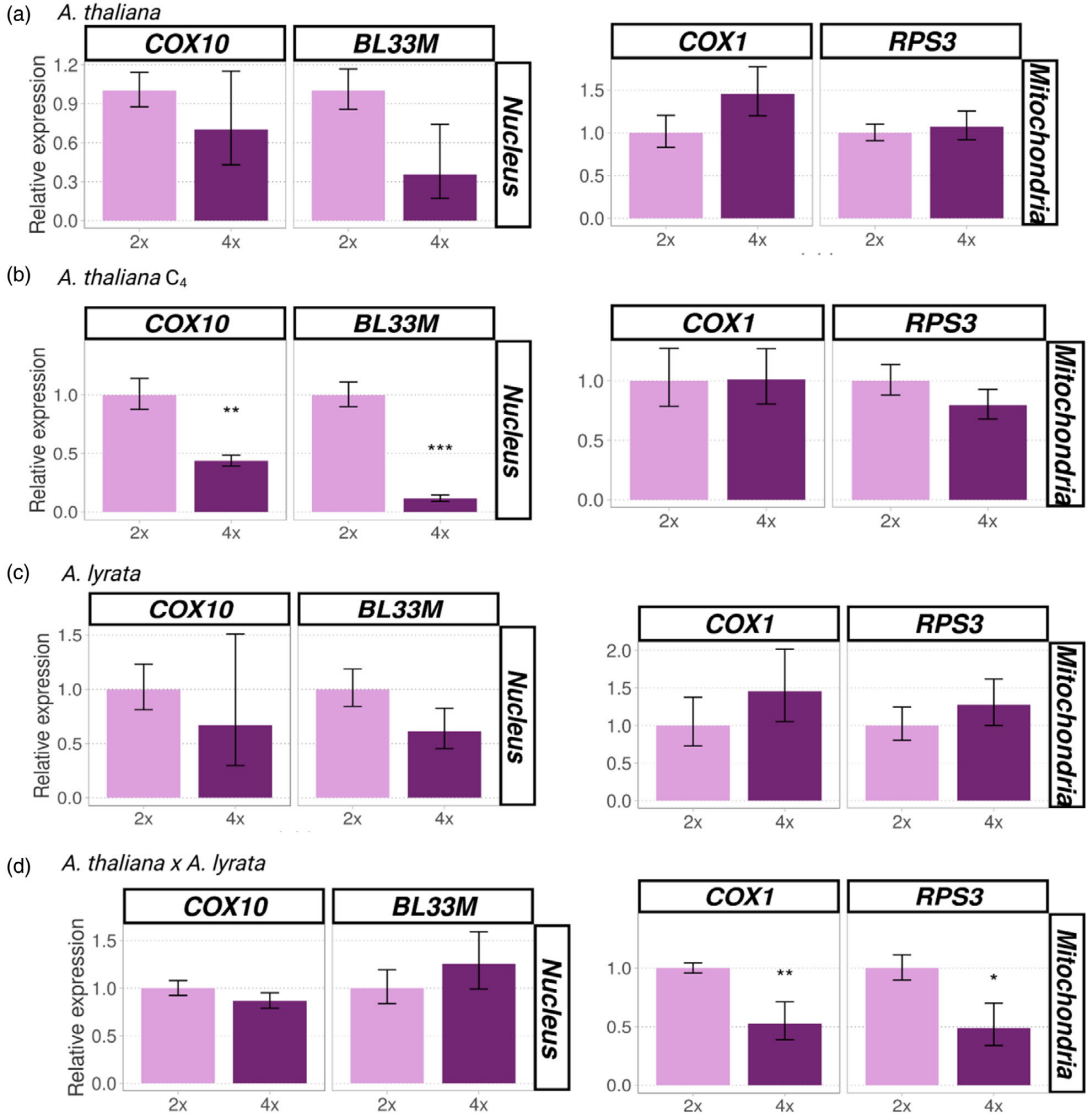
Changes in expression of cytonuclear genes involved in mitochondrial complexes after WGD in leaves of *Arabidopsis thaliana, Arabidopsis lyrata*, and *A. thaliana* × *A. lyrata* hybrids. (a–d) Each plot represents the relative transcript abundance (*y* axis) of nuclear‐encoded (left) and mitochondria encoded genes (right) involved in cytonuclear complexes in diploid (pink) and corresponding polyploid (purple) plants based on qRT‐PCR in *A. thaliana* C1 (a), *A. thaliana* C4 (b), *A. lyrata* (c) and *A. thaliana* × *A. lyrata* (d). Error bars represent the standard error of the mean of the biological replicates (*n* = 3–4). ***P < 0.001, **P < 0.01, *P < 0.05 (based on two‐tailed Student's *t*‐test).

The results showed a consistent qualitative decrease in transcription levels of nuclear genes in autopolyploids of *A. thaliana* and *A. lyrata* compared to their diploid progenitors (Figures 6a–c and 7a–c), even though a statistically significant decrease was observed only in the C_4_ generation of autotetraploid *A. thaliana* with a much higher reduction of transcript levels of nuclear genes involved in mitochondria‐nuclear complexes (*COX10*, *P* = 0.0011; *BL33M*, *P* < 0.001; Figure 7b) than those involved in chloroplast‐nuclear complexes (*ATPC1*, *P* = 0.260; *PETC*, *P =* 0.047; *PSBR*, *P* = 0.003; Figure 6b). The transcript levels of organellar genes involved in cytonuclear complexes varied: there was a general, though non‐significant, increase in autopolyploid organellar gene expression compared to that found in diploids (Figures 6a–c and 7a–c), except for transcription of chloroplast and mitochondrial genes, which was the same or slightly lower in the C_4_ generation of *A. thaliana* autotetraploid (Figures 6b and 7b).

The changes in gene expression patterns following WGD appear to be quite different in allopolyploids compared to autopolyploids. In particular, chloroplast genes were transcribed at significantly higher levels in allotetraploid *A. thaliana* × *A. lyrata* compared to the diploid hybrid (*ATPI*, *P* < 0.001; *PETA*, *P* = 0.003; *PSBD*, *P* = 0.038), but we observed no change in relative expression of nuclear genes involved in chloroplast‐nuclear complexes, indicating that the elevated gene dosage contributes to elevated transcript dosage balance, at least for chloroplast genes (Figure 6d). To confirm these observations, analyses were repeated after two successive generations of allopolyploids. The same pattern of upregulation was observed in the third generation of allopolyploids (*ATPI*, *P* = 0.036; *PETA*, *P =* 0.035; *PSBD*, *P =* 0.029; Figure S3). In contrast, mitochondrial genes showed a statistically significant decrease in transcript levels in allopolyploids relative to diploid hybrids (*RPS3*, *P* = 0.032; *COX1*, *P* = 0.007), with no change in the transcription of the corresponding nuclear genes (Figure 7d).

Overall, our results suggest that WGD had little immediate effect on transcription of genes involved in cytonuclear interactions in newly developed autotetraploids, but that expression decreased in subsequent generations, especially for nuclear genes involved in mitochondria‐nuclear complexes. In contrast, an immediate response to WGD was apparent in allotetraploids. Transcription levels of organellar genes were significantly altered in the first generation, but genes from the two organelles responded in opposite ways: transcription was reduced for mitochondrial genes, but increased for chloroplast genes. Overall, we observed two main patterns for gene expression: (1) downregulation of nuclear genes involved in both chloroplast‐nuclear and mitochondria‐nuclear complexes in the C_4_ generation of autopolyploid *A. thaliana* and (2) upregulation of chloroplast and downregulation of mitochondrial genes involved in cytonuclear complexes in allopolyploids.

To explore the relationships of expression to their function in cytonuclear complexes, we analyzed gene expression of organelle‐targeted nuclear genes and organellar genes not involved in cytonuclear complexes. In most cases, there were no statistically significant differences in relative expression levels between diploids and tetraploids (Figure S4). This indicates that organelle‐targeted nuclear genes and organellar genes not involved in cytonuclear complexes are less sensitive to stoichiometric imbalances caused by WGD. There was a single exception: chloroplast genes not involved in cytonuclear complexes were upregulated in allotetraploids compared to diploid hybrids (*RPOC2*, *P* = 0.005; *RPOB*, *P* = 0.042), similar to the pattern observed in chloroplast genes involved in cytonuclear enzyme complexes (Figure S4b).

As an alternative complementary approach, we reanalyzed our qPCR data using the raw Cq values to calculate ΔCq for each organelle–nuclear partner pair (see Experimental Procedures). Across all datasets, organelle: nuclear stoichiometric ratios were either maintained or increased in tetraploid relative to diploid plants. In chloroplast complexes, *PSBD*/*PSBR* and *PETA*/*PETC* ratios showed a significant increase in C_4_ generation of *A. thaliana* (Figure S5). The same pattern was observed for the mitochondria *RPS3*/*BL33M* (both C_1_ and C_4_ generations in *A. thaliana*) and *COX1*/*COX10* (C_1_ generation in *A. thaliana*). The results support the conclusion that cytonuclear transcript balance is maintained or biased towards organelle transcripts in tetraploid plants.

## DISCUSSION

Cytonuclear interactions are at the heart of the maintenance of key physiological functions within eukaryotic cells, including photosynthesis and aerobic respiration. These interactions and the coevolution of the nuclear and organellar genome play an important role in the evolution of plant species (Kan et al., 2024; Roux et al., 2016; Sharbrough et al., 2017; Sloan et al., 2018). Disruptions to well‐tuned stoichiometry between nucleus‐encoded and organelle‐encoded proteins involved in cytonuclear complexes can lead to mitochondria and chloroplast dysfunction and developmental defects (Burton et al., 2013; Havird et al., 2019). Such disruption can be caused by WGD, a frequent event in plant evolution. While nuclear genes are doubled in WGD, this is not immediately followed by doubling the number of organelles. Various mechanisms are sometimes employed to adjust stoichiometry (Bingham, 1968; Krishnaswami & Andal, 1978; Shahbazi et al., 2024), and reply to the altered ratio between organellar and nuclear genomes. Here, we investigated the dynamics of the stoichiometry response in sequential generations, comparing auto‐ and allopolyploids, and for two different plant organelles, mitochondria and chloroplasts. All polyploids were developed using colchicine treatment. While this represents an unprecedented opportunity to evaluate the instant reaction of the plants to whole‐genome duplication, it also brings possible concerns about the additional effects of colchicine. It was found that colchicine might affect cell morphology (Dolezel & Binarova, 1989; Tammu et al., 2021) and gene expression (Bhuvaneswari et al., 2020). Another potential bias could be represented by endoreduplication. There is not a simple way to distinguish endoreduplicated cells from non‐endoreduplicated cells morphologically. Higher ploidy should be linked with larger volumes of the cells; however, the variation in cell volume even in diploid plants with low (or no) endoreduplication shows that this is a highly challenging task [Fernandes Gyorfy et al. (2021) Plant J doi: 10.1111/tpj.15436; Lim et al. (2020) Gene Rep doi: 10.1016/j.genrep.2020.100808; Preuten et al. (2010) Plant J doi: 10.1111/j.1365‐313X.2010.04389.x]. We mitigated the potential bias caused by endoreduplication in genome copy number and expression analyses using normalization per nuclear genome copy (rather than per ploidy) and housekeeping gene expression, respectively. Last, but not least, the results can be biased by the differences in the relative extraction efficiency for organelle DNA relative to nuclear DNA, or any confounding of this extraction ratio based on cell size/volume. To mitigate this risk, we used 3–5 biological and at least two technical replicates for all analyses.

### Auto‐ and allopolyploids respond differently to WGD

**Figure 8:**
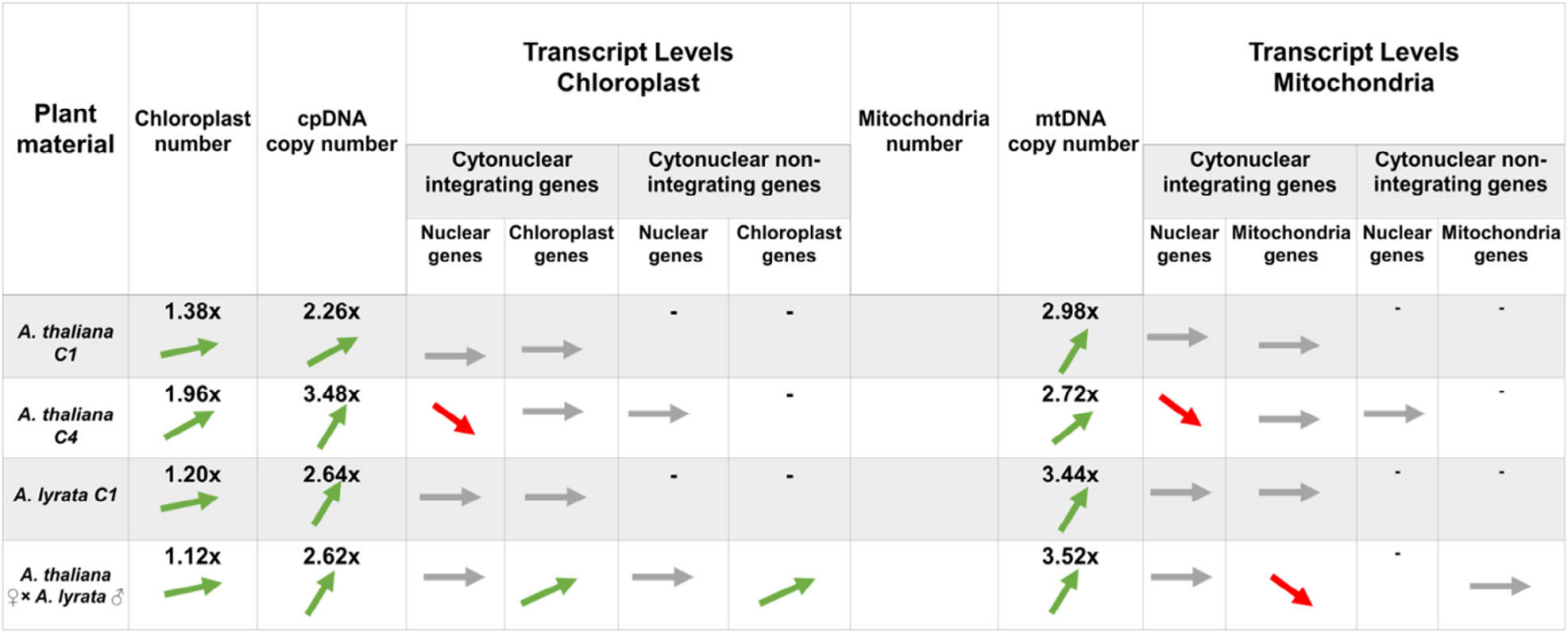
Overview of the mechanisms involved in cytonuclear balance restoration in *Arabidopsis thaliana, Arabidopsis lyrata*, and their hybrids. The figure presents fold change differences in organelle number (per diploid and tetraploid cell), genome copy number (per diploid and tetraploid cell), and changes in gene expression of cytonuclear interacting and non‐interacting genes across different ploidy levels (per diploid and tetraploid cell). Green arrows indicate an increase, and red arrows indicate a decrease.

In autopolyploids, the stoichiometric adjustment accompanying polyploidy is mainly triggered by an increase of cpDNA and mtDNA copies (Coate et al., 2020; Fernandes Gyorfy et al., 2021; Shahbazi et al., 2024). Fernandes Gyorfy et al. (2021) and Shahbazi et al. (2024) demonstrated full compensation (i.e., at least doubling of the number of organellar genome copies) in synthetically derived autotetraploids. Shahbazi et al. (2024) observed a 2.1‐fold increase in the number of cpDNA copies in the first generation (C_1_) of autotetraploid *F. pratensis*. Fernandes Gyorfy et al. (2021) showed doubling of both the cpDNA and mtDNA copies with each doubling of the nuclear ploidy in the third generation of synthetically derived tetraploid and octoploid *A. thaliana*, while Coate et al. (2020) found full compensation of mtDNA and only partial compensation of cpDNA in synthetic autotetraploid *A. thaliana*. Our study demonstrates overcompensation in newly developed autotetraploid *A. thaliana* and *A. lyrata*, with cpDNA copies increasing by 2.26‐fold and 2.64‐fold and mtDNA copies by 2.98‐fold and 3.44‐fold, respectively. These increases were not proportional to the number of organelles, for which numbers increased only by 1.38‐fold (*A. thaliana* chloroplasts), 1.2‐fold (*A. lyrata* chloroplasts) and 1.29‐fold (*A. thaliana* mitochondria), respectively (Figure 8). As a result, each organelle in autopolyploids appears to have more copies of DNA than in diploids.

A similar increase in chloroplast numbers (1.38‐fold) was previously found in mesophyll cells and a slightly higher increase (1.5‐fold) in the subepidermal palisade cells of autotetraploid *A. thaliana* (He et al., 2021; Kawade et al., 2013). Oberprieler et al. (2019) found a similar pattern in the increase of chloroplasts and chloroplast genomes in closely related diploid, tetraploid, and hexaploid species of *Leucanthemum*. The relationship between the WGD and the number and size of mitochondria has not been previously studied. However, Preuten et al. (2010) reported a linear increase in the number of mitochondria with the increasing cell size in Arabidopsis protoplasts, suggesting a similar trend after WGD, given that an increase in cell size often accompanies polyploidy.

Despite changes in the organelle genome copy numbers, transcription of nuclear and organellar genes involved in cytonuclear complexes remained stable across ploidy levels, at least for the small sampling of genes studied here. We did not detect any significant changes in transcript levels in early‐generation autopolyploids (Figure 8). This is in line with observations of Forsythe et al. (2022) and Shahbazi et al. (2024). This stability may suggest some initial buffering mechanism (Ma et al., 2025). In later generations (C_4_) of *A. thaliana* autotetraploids, we detected a pronounced downregulation of nuclear genes involved in both chloroplast and mitochondrial cytonuclear complexes, and also some reduction in mtDNA copy number; the pattern was observed also in other studies (Chen, 2007; Guo et al., 1996). Because this downregulation arose within selfed lines, we expect that it has an underlying epigenetic basis.

Allopolyploids, formed by the merger of divergent genomes, face an additional layer of complexity in nuclear‐organelle incompatibilities. Since nuclear genes are inherited biparentally but organelles are inherited maternally in the majority of species (including *Arabidopsis*; Zouros, 2013, 2020), hybridization may lead to cytonuclear incompatibilities due to sequence divergence between either organellar or nuclear copies of genes. Genetic differences between the nuclear homoeologues can potentially cause genetic incompatibilities when interacting with an organellar counterpart originating from the other parent (Sharbrough et al., 2017; Sloan et al., 2024). If not resolved, these incompatibilities can lead to organelle dysfunction and even to hybrid breakdown (reviewed in Burton et al., 2013). It has been observed that nuclear genes involved in cytonuclear complexes often return to the single copy status after WGD (recently reviewed by Sloan et al., 2024), and that paternal homoeologues may rapidly evolve or be eliminated (de Smet et al., 2013), although no global evidence for this pattern has yet been found (de Carvalho et al., 2019; Sharbrough et al., 2022). Both maternal expression level dominance and a bias toward expression of the maternal homoeologue have also been detected in allopolyploids (de Carvalho et al., 2019; Gong et al., 2014; Grover et al., 2022; Wang et al., 2017; Zhai et al., 2019; Zhang et al., 2023).

In newly established allopolyploids, adjustments to gene expression can be highly flexible. Unlike autopolyploids, *A. thaliana* × *A. lyrata* allopolyploids showed immediate modifications of gene expression after WGD. Although there were no statistically significant changes in the overall transcript levels of nuclear genes involved in cytonuclear complexes, chloroplast genes were upregulated and mitochondrial genes were downregulated relative to diploids. This suggests that a merger of distinct genomes affects not only biased transcription of nucleus‐encoded homoeologues but possibly also a mechanism of stoichiometry adjustments after WGD. Changes to regulatory elements have previously been reported to drive selection on stoichiometric balance after polyploidization (Gillard et al., 2021). Our data demonstrate that autopolyploids and allopolyploids show different responses to WGD. Although the change in organelle genome copy numbers is similar to the autopolyploids, allopolyploids show a considerably smaller increase in the number of chloroplasts (1.12‐fold) relative to their corresponding diploids. Allopolyploid chloroplasts have fewer genome copies than those of autopolyploids. Although the responsible mechanism for this difference is not known, the correlation with divergence (allo vs. auto) implies regulatory disruption of the replication machinery brought about by the presence of two homoeologues of nuclear genes involved in chloroplast regulation and multiplication. Additionally, for the several genes studied here, allopolyploids show an instant modification of gene expression—upregulation of chloroplast genes and downregulation of mitochondrial genes. In autopolyploids, changes in gene expression were observed only in the C_4_ generation, exclusively affecting nuclear genes (downregulation; Figure 8). The mechanisms behind these differences remain unknown.

### Instant response to WGD is followed by dynamic adjustments in successive generations

WGD triggers an immediate increase in organellar genome copies (reviewed in Sloan et al., 2024). But is this immediate response followed by further adjustments? Is the overcompensation (over two‐fold increase in the number of cpDNA and mtDNA copies) seen here and in other studies ultimately scaled down to simply double the organellar genome copies relative to diploids? Our results suggest a dynamic adjustment. For mtDNA in *A. thaliana*, the initial 2.98‐fold increase (per nuclear genome copy ratios; see Results) in C_1_ declines to 2.72‐fold by C_4_. In contrast, cpDNA copies continue rising, from 2.30‐fold in C_1_ to 3.48‐fold in C_4_ (Figure 8). A similar pattern appears in *F. pratensis*, where cpDNA copies increase from 2.1‐fold in C_1_ to 2.7‐fold in C_6–8_ (Shahbazi et al., 2024). The increase in organelle genome copy number is consistent with the dynamics of organelle number in polyploid cells. Organelle numbers also continue to increase in subsequent generations of polyploids. While the organelle numbers and genome copies respond immediately, in our study gene expression changes occur later. Transcript levels remained stable in C_1_ but were altered in C_4_, suggesting a delayed regulatory response to WGD.

For nuclear genes involved in cytonuclear complexes, the return to a diploid‐like state has been considered a possible evolutionary response to WGD. However, the contradictory results from different plant models suggest that this may be species‐ and gene‐specific (Sharbrough et al., 2022; Sloan et al., 2024). In allopolyploid evolution, preferential elimination of paternal alleles, paternal‐to‐maternal gene conversion, and biased gene expression favoring maternal homoeologues have been observed and also hypothesized to be widespread and important (de Carvalho et al., 2019; Gong et al., 2014; Grover et al., 2012; Mason & Wendel, 2020; Ortiz & Sharbrough, 2024; and reviewed in Sloan et al., 2024). Yet even these processes seem to be taxon‐specific (Grover et al., 2022; Sharbrough et al., 2022).

### Mitochondria and chloroplasts display different responses to WGD

Although chloroplasts and mitochondria retain their own genomes containing protein‐coding genes, they are both regulated by the nucleus via anterograde (nucleus‐to‐organelle) and retrograde (organelle‐to‐nucleus) signaling. Expression of organellar genes is mostly regulated by nucleus‐encoded transcription factors, with the exception of the chloroplast‐encoded RNA polymerase (reviewed in Woodson & Chory, 2008; Jan et al., 2022).

Our results suggest that mitochondria and chloroplasts respond differently to WGD and/or hybridization. The increase in the organelle genome copy number seems to be a general response to polyploidization, but there are exceptions. Fernandes Gyorfy et al. (2021) found that hexaploid wheat *T. aestivum* had a higher number of cpDNA copies, but not mtDNA copies, compared to the tetraploid *T. turgidum*, which may be due to overcompensation by the tetraploid. Furthermore, they found that dihaploids generated from autotetraploids of *A. thaliana* showed a significant decrease in the number of mtDNA copies compared to their tetraploid progenitor (1:1.65 ratio), but the number of cpDNA copies decreased only marginally (1:1.14). In our study, we found that in newly created polyploids, the numbers of mtDNA copies increased more than the numbers of cpDNA copies (despite slightly higher increase of number of chloroplasts than number of mitochondria: 1.38‐fold vs. 1.29‐fold). However, the number of cpDNA copies increased further in the C_4_ generation of *A. thaliana*, while the number of mtDNA copies dropped (Figure 8). This may indicate either a higher need for increased cpDNA copy number and/or more relaxed selection for the disequilibrium between nuclear and organellar counterparts in chloroplasts than in mitochondria. The mechanisms responsible for these observations and differences are unknown, as is the answer to the question of whether they have functional significance.

The differences in genomic copy numbers are more pronounced at the level of transcription. Comparative RNAseq analysis of autopolyploid versus diploid *A. thaliana* revealed that both mitochondrial and nuclear subunits interacting in mitochondrial complexes showed stable expression, or coordinated upregulation, in tetraploids versus diploids, whereas both chloroplast and nuclear genes involved in chloroplast complexes were downregulated (Coate et al., 2020). Here, we found an opposite trend in transcript levels in response to WGD, especially in allopolyploids. Chloroplast genes involved in cytonuclear interactions showed a significant upregulation in tetraploids relative to diploids, whereas mitochondrial genes were significantly downregulated. With the proviso that relatively few genes were studied, upregulation of transcription appeared to be a general trend across the chloroplast genome following hybridization and polyploidization, whereas downregulation of mitochondrial genes was restricted to those that directly interact in cytonuclear complexes (Figure 8). This indicates that after WGD in hybrids, chloroplast gene expression undergoes global reprogramming, while mitochondrial gene expression is fine‐tuned to compensate for imbalance in the ratio of nuclear and mitochondrial subunits (Figure 8). Since organelle regulation primarily occurs at the posttranscriptional level (del Campo, 2009; Forsythe et al., 2022; Jung et al., 2013), it would be worthwhile to compare transcript‐level changes with corresponding protein levels to better understand the regulatory mechanisms involved. There might be differences in protein translation, import, protein folding, and assembly (Sloan et al., 2024). Another factor could be differences in the overall abundance of the organellar transcripts. It has been shown that chloroplast transcripts account for 60–80% of the total mRNA pool, while mitochondrial transcripts account for only 1–4% (Forsythe et al., 2022).

Accumulating data suggests that, in general, there may be differential responses of chloroplast and mitochondrial genomes to WGD. In two *Brassica* allopolyploids, nuclear genes targeted to chloroplasts experienced selection to match corresponding chloroplast genomes, whereas nuclear genes targeting mitochondria did not show such selection (Kan et al., 2024). Similarly, chloroplast‐targeted genes exhibit maternally biased homoeologous conversion, while mitochondrial genes exhibit a dearth of homoeologous recombination in *Coffea arabica* (Ortiz & Sharbrough, 2024). Due to maternally inherited organellar genomes, allopolyploids are expected to retain maternal copies of the nuclear genes involved in cytonuclear complexes more frequently than paternal copies, but it is not always the case (Camus et al., 2022; Gong et al., 2012, 2014; Kan et al., 2024; Sharbrough et al., 2017; reviewed in Sloan et al., 2024).

### Polyploids have smaller chloroplasts than diploids, while mitochondria have the same volume

Another intriguing feature is a decrease in the volume of chloroplasts in polyploids. Size of the chloroplast compartment is known to be cell type specific (Larkin et al., 2016; Osteryoung & Pyke, 2014). In general, higher numbers of smaller chloroplasts appear to be evolutionarily preferred over a lower number of larger chloroplasts (Xiong et al., 2017). This might be related to aspects of nitrogen‐use efficiency, lower specific activity of RuBisCo in larger chloroplasts, more efficient chloroplast movement, more efficient photosynthesis and photoprotection in smaller chloroplasts, or other factors (Coate et al., 2012; Ilut et al., 2012; Jeong et al., 2002; Vyas et al., 2007). Here we found that the average volume of chloroplasts was smaller in both auto‐ and allopolyploids relative to their diploid parents. Similarly, we found that autotetraploid cultivars of *L. multiflorum* had about 10% smaller chloroplasts than diploid ones (Shahbazi et al., 2024). In contrast, tetraploids of a closely related species, *F. pratensis*, have a slightly higher average volume of chloroplasts (Shahbazi et al., 2024). Even in this case, however, we see a trend towards a reduction in the volume of chloroplasts in subsequent generations of polyploids.

In summary, both auto‐ and allopolyploids respond to WGD immediately by increasing organelle genome copies, partially accomplished by increasing the number of organelles. Allopolyploids also immediately adjust organellar gene expression. In contrast, autopolyploids showed gene expression changes only in later generations and, unlike in allopolyploids, these changes involved nuclear genes, potentially indicating that retrograde communication is more efficient in autopolyploids than in allopolyploids. Mitochondria and chloroplasts differ in their reactions to WGD. Chloroplasts appear to revert more quickly to the ancestral, diploid‐like condition, especially in allopolyploids, perhaps due to the incompatibility of paternally inherited nuclear genes with maternally inherited organellar counterparts (Coate et al., 2020; Emery et al., 2018; Fernandes Gyorfy et al., 2021; this study).

Overall, this and earlier studies indicate that WGD in plants initiates an immediate cytonuclear set of responses, but the imbalance between nuclear and organellar counterparts does not appear to significantly affect viability and fertility (Prost‐Boxoen et al., 2024). There likely are several explanations for this generalization. First, all studies are conducted on ‘winners’, meaning that the most physiologically disturbed scenarios caused by stoichiometric imbalance between nuclear and organelle components of the chimeric protein complexes may not survive to be studied. Second, in many plant species, including *Arabidopsis*, endoreduplication (doubling of the nuclear genome without cell division or mitosis) is common in at least some organs or tissues and hence, indicates that at least some cells and tissues can leverage pre‐existing molecular machinery to accommodate altered ratios between organelle and nuclear genomes (Kawade et al., 2013; Pacey et al., 2020). To the extent that this is true, tolerance of altered cytonuclear stoichiometries may be part of a widespread physiological condition in plants.

## EXPERIMENTAL PROCEDURES

### Plant material

All experiments described in this study were carried out on leaf material from diploid and tetraploid *A. thaliana* Col‐0, *A. lyrata* MN47, and their hybrids (*A. thaliana* × *A. lyrata*). Leaves of *A. thaliana* and the hybrids were harvested from 1‐month‐old plants, whereas leaves of *A. lyrata* were, due to slower growth, harvested from 2‐month‐old plants (i.e., at a similar developmental stage). Fully expanded leaves were collected for all experiments at the same time and from the same (first) layer of rosettes to ensure comparability. Three to six biological replicates (individual plants) were analyzed in all experiments. Tetraploid lines were generated from the six diploids by colchicine treatment as follows: Seedlings grown on 1/2 Murashige and Skoog medium (Duchefa Biochemie) with the first true leaf pair developed were submerged in 0.1% colchicine and kept in the dark for 2 h. The seedlings were then thoroughly washed in water and planted in soil (Klasmann‐Deilmann GmbH Substrate 1 + GreenFibre®) in 5 cm^2^ pots. Plants were kept under controlled conditions: 16 h light, 8 h dark, 20°C during the light phase and 18°C during the dark phase of the cycle, and 60% humidity. Six confirmed tetraploids (C_0_) were selfed to produce C_1_ seed. In the case of *A. thaliana*, we produced the next three generations from these six C_1_ plants of autotetraploids and the plants of the C_4_ generation were also used in our analyses.

### Flow cytometry

Ploidy levels of all plants were estimated according to Dolezel et al. (2007) in nuclear suspensions prepared from 50 mg of leaf tissue. Samples were analyzed using a CyFlow Space flow cytometer (Sysmex Partec GmbH., Görlitz, Germany) equipped with a UV LED diode array. At least 5000 events were acquired per sample, and only measurements with the coefficient of variation for G0/G1 peaks <2.0% were accepted.

### Morphological characters

Chloroplast numbers and volumes were assessed in mesophyll cells using cryotome sections as in Shahbazi et al. (2024). A Leica TCS SP8 confocal microscope equipped with Leica Application Suite X (LAS‐X) v.3.5.5 software and Leica Lightning module (Leica Microsystems) was used for imaging. Generation of 3D models of microscopic images and volume calculations were performed using IMARIS 9.7 software (Bitplane AG, Schlieren, Switzerland). IMARIS functions ‘Surface’ and ‘Spot Detection’ were used for the manual analysis of each cell. Chloroplast volume was determined from the rendering of primary intensity of autofluorescence (emission wavelengths 650–700 nm) while using the ‘Surfaces’ function, and subsequently using statistical function ‘Volume’. Chloroplast numbers were counted manually in each cell. 20–30 cells and 20–30 chloroplasts were analyzed in each plant (~100 measurements per ploidy). We used six, six, and six plants of *A. thaliana* (diploid, C_1_ tetraploid and C_4_ tetraploid), four and three plants of *A. lyrata* (diploid and tetraploid) and six and three plants of *A. thaliana* × *A. lyrata* (diploid and tetraploid) for chloroplast number and volume measurements.

To estimate changes in the number and volume of mitochondria in diploid versus tetraploid *A. thaliana*, we used a publicly available mitochondria CFP marker line, mt‐ck (NASC number: N16262). Initially, we stained the leaves with the mitochondria marker dye Mitotracker® Red CMXRos (ThermoFisher Scientific) to confirm that CFP and Mitotracker colocalize in the mitochondria. Staining followed the manufacturer's instructions: the leaf tissue was first submerged in 0.2% Triton X‐100 in 1x PBS for 10 min, then transferred into a 0.3 μM Mitotracker® Red CMXRos solution in ½ MS media (Duchefa). The samples were vacuum infiltrated twice for 15 min, then incubated in the dye overnight in the dark at 4°C. Following this, samples were mounted in ½ MS media with 10 μL Vectashield (Vector Laboratories, Inc.) and imaged using a Leica confocal microscope, as described in the chloroplast analysis. For fluorophore excitation, we used the following lasers: CFP at 405 nm, MitoTracker at 578 nm, and chlorophyll at 633 nm. Emission was captured with the following filters: 412–501 nm bandpass for CFP, 586–613 nm bandpass for MitoTracker, and a 640 nm long pass for chlorophyll. Colocalization of CFP and Mitotracker was confirmed, but penetration of Mitotracker into the cells was not uniform and thus did not stain all mitochondria (Figure S2). Therefore, all subsequent imaging was conducted on the mt‐ck line, which was much more consistent. We acquired ten Z‐stack images per sample (five biological replicates for diploids and five for tetraploids) using a 63× immersion objective lens and analyzed the data using IMARIS software. Given the large number of mitochondria per cell and their dynamic behavior with frequent fusions and fissions, individual mitochondria were not counted. Instead, we assessed the total number and volume of fluorescent foci per constant Z‐stack volume (185 × 185 × 15 μm) automatically using functions ‘Surface’ and ‘Spot Detection’ to generate a 3D model of the organelle (Background subtraction set to 1 um and Surface detail set to 0.3 um). We used five and five plants of *A. thaliana* (diploid and C_1_ tetraploid) for mitochondria number and volume estimations.

A two‐sample, two‐tailed *t*‐test was used to compare means between groups. To determine the magnitude of the difference between the groups, the effect size was calculated using Cohen's *d* test with classification as follows: negligible effect *d* < 0.2; small effect *d* = 0.2– 0.5; moderate effect *d* =0.5–0.8; large effect *d* ≥ 0.8 (Sullivan & Feinn, 2012). Data analysis was performed using RStudio, and data were visualized using ggplot2.

### Organelle genome copy number counting using droplet digital PCR (ddPCR)

For the ddPCR experiment (as well as for qPCR experiment; see below), we used four, six, and six plants of *A. thaliana* (diploid, C_1_ tetraploid and C_4_ tetraploid), four and three plants of *A. lyrata* (diploid and tetraploid) and six and three plants of *A. thaliana* × *A. lyrata* (diploid and tetraploid) for chloroplast number and volume measurements. Genomic DNA was isolated from the leaves and digested with *Hin*dIII‐HF (NEB) restriction enzyme for 16 h to increase the efficiency of the subsequent amplification process. A 20 μL ddPCR reaction consisted of 10 μL QX200 ddPCR EvaGreen Supermix (Bio‐Rad, Hercules, CA, USA), 100 nM forward and reverse primers, and a template concentration of either 0.01 ng μL^−1^ (for chloroplast genes) or 0.1 ng μL^−1^ (for mitochondrial genes), or 0.5 ng μL^−1^ for nuclear genes. Plates were sealed using the PX1 PCR Plate Sealer (Bio‐Rad, Hercules, CA, USA). Amplification of genes of interest was performed for 5 min at 95°C, followed by 40 cycles of 30 sec at 95°C and 1 min at 58°C. Final signal stabilization was performed for 5 min at 4°C and 5 min at 90°C. The ramp rate was set at 2°C/sec for each temperature change. A QX200 Droplet Reader (Bio‐Rad, Hercules, CA, USA) was used to measure positive droplets and calculate copy numbers in each sample. Quantasoft v1.7 software (Bio‐Rad, Hercules, CA, USA) was used to calculate copy numbers using Poisson statistics. Two single‐copy nuclear genes, photosystem II subunit R (*PSBR*) and ATP synthase subunit beta‐1 (*ATPβ1*) were used to determine the copy number of the nuclear genome in each well. Chloroplast gene NAD(P)H dehydrogenase subunit B (*NDHB*), and mitochondrial gene ATP synthase membrane subunit 6 (*ATP6*), both in two copies within the genome, were selected as controls. Their copy numbers should be twice as high as those of the single‐copy genes and therefore were divided by 2 in the analysis.

To determine the chloroplast and mitochondrial genome copy number per nuclear genome copy, we first divided the copy number of organelle‐encoded genes (after adjusting for the dilution factor) by the number of nuclear genome copies (given by the number of *PSBR* and *ATPβ1* copies in each sample) in both diploid and tetraploid samples. *PSBR* (involved in chloroplast‐nuclear complex) was used for chloroplast genome copy number estimates, while *ATPβ1* (involved in mitochondria–nuclear complex) was used for estimating mitochondria genome copy numbers. We then calculated the stoichiometric compensation ratio by dividing the organelle genome copy number in tetraploid samples by the genome copy number in diploid samples. Similar ratios indicate compensation (*P*‐value >0.05), ratios greater than one are considered indicative of overcompensation (*P*‐value <0.05), while those less than one signify undercompensation (*P*‐value <0.05). The primers used in these experiments are listed in (Table S1). Data analysis was performed using RStudio, and data were visualized using ggplot2. A linear mixed‐effects model was used as in Fernandes Gyorfy et al. (2021) to assess whether gene, ploidy, and their interaction were significant predictors of mtDNA or cpDNA copy numbers per nuclear genome copy. The model was fit using the lmer function in the lme4 R package (Bates et al., 2015). Fixed effects in the model include gene, ploidy, and gene/ploidy interaction, while the random effect is individual, accounting for variability across biological samples. The Analysis of Deviance was performed using the anova function with Type III sums‐of‐squares from the car package (Fox & Weisberg, 2019). Post‐hoc pairwise comparisons were conducted using the emmeans function from the emmeans package (Lenth, 2024).

### Gene expression analysis

For the qRT‐PCR experiment, total RNA was isolated from diploid and tetraploid plants of *A. thaliana*, *A. lyrata*, and *A. thaliana* × *A. lyrata* (see above; additionally, we used three plants of F_3_ generation of *A. thaliana* × *A. lyrata* generated from three individual plants of F_1_ generation) using the RNeasy Kit (QIAGEN). The quality and quantity of the samples were assessed using a Qubit Fluorometer (Life Technologies), a Nanodrop ND‐1000 spectrophotometer (Nanodrop Technologies, USA), and 1% agarose gel electrophoresis. After DNase I (Thermo Scientific™) treatment of 500 ng of RNA, cDNA synthesis was performed using the RevertAid First Strand cDNA Synthesis Kit (Thermo Scientific™) with random hexamer primers to reverse transcribe both organelle‐ and nuclear‐encoded transcripts. The qRT‐PCR experiment was conducted on a CFX96 Real‐Time PCR Detection System (Bio‐Rad, USA). Primer efficiency was evaluated by generating standard curves using a 5‐point, 10‐fold serial dilution series of cDNA. The slope (m) of the standard curve was used to calculate the primer amplification efficiency (E) using Maestro software v2.0 (Bio‐Rad, USA). Only primers with efficiencies between 90% and 110% were considered acceptable. The qRT‐PCR cycling program consisted of an initial denaturation step at 94°C for 2 min, followed by 40 cycles of denaturation at 94°C for 15 sec, annealing at 58°C for 15 sec, and extension at 72°C for 20 sec. To confirm the specificity of the amplified products, a melting curve analysis was conducted by ramping the temperature from 65 to 95°C. Transcript‐level changes were analyzed using the ΔΔCt method (Livak & Schmittgen, 2001) and Maestro software v2.0 (Bio‐Rad, USA). To normalize the gene expression data, the maturase K (*MATK*) and intron maturase, type II (*MATR*) genes were selected as reference genes for chloroplasts and mitochondria, respectively. Conversely, the ACTIN 2 (*ACT2*) and eukaryotic initiation factor 4A (*EIF4A*) were selected as the reference for the nuclear genes (Huang et al., 2014; Lechowicz et al., 2020; Shahbazi et al., 2024; Wang et al., 2014). We used *ACT2* as a primary reference gene for all the qPCR experiments and confirmed the results using *the EIF4A* gene in additional qPCR experiments (for selected qPCR runs). As an alternative approach, we re‐analyzed our qPCR data using the raw Cq values to calculate ΔCq for each organelle–nuclear partner pair. For each pair, we computed:

$$\begin{array}{c} \mathrm{\Delta Cq}=Cq\;\text{organelle}\;-\;Cq\;\text{nuclear},\\ \;\text{log2}\left(\text{ratio}\right)=-\mathrm{\Delta Cq} \end{array}$$

This allowed us to estimate the relative stoichiometric ratio of organelle to nuclear transcripts for each ploidy level. We then compared the ratios between diploid and tetraploid plants and calculated fold changes with 95% confidence intervals. An unpaired (independent samples) two‐tailed Student's *t*‐test was employed for comparisons between diploids and tetraploids. All primers used in the qRT‐PCR experiment are listed in (Table S1). Data were visualized using ggplot2.

## AUTHOR CONTRIBUTIONS

DK conceived the project; JK developed all polyploids; MSh, JK and DK developed the methodology. MSh, JK, DKu, AD, MSz and YDS conducted the study and processed the data; MSh, JK and DKo wrote the original draft; JFW, JS and MSz reviewed and edited the manuscript; DK acquired the funding. All authors have read and agreed to the published version of the manuscript. MSh and JK contributed equally to this work.

## CONFLICT OF INTEREST

The authors declare no conflict of interest.

## Supporting information


**Data S1.** Chloroplast/mitochondria number and volume quantification (Figures 2 and 3) in *A. thaliana* (At), *A. lyrata* (Al) and *A. thaliana × A. lyrata* (At × Al) diploids (2×) and tetraploids (4×). Number of chloroplasts was counted per cell, and number and total volume of mitochondria were calculated per Z stack image (see Experimental Procedures for details).

**Data S2.** cpDNA and mtDNA copy number analysis (Figures 4 and 5). Digital droplet PCR analysis of the number of chloroplast and mitochondria genome copies based on three and two chloroplast and mitochondria genes, respectively, involved in cytonuclear complexes. Values were normalized per nuclear genome copy, that is, divided by the number of copies of the nuclear gene (PSBR and ATPβ1 for chloroplasts and mitochondria respectively) in the sample.

**Data S3.** Linear mixed‐effect model output (Figures 4 and 5) was fit using the lmer function in the lme4 R package. Fixed effects are gene, ploidy, and gene/ploidy interaction. The random effect is an individual. The analysis of Deviance was performed using the Anova function with Type III sums of squares from the car package. Post hoc pairwise comparisons were conducted using the emmeans function from the emmeans package.

**Data S4.** Transcript‐level analysis (Figures 6 and 7). qPCR analysis of transcript levels of selected organelle genes and their interacting nuclear counterparts as well as non‐interacting organelle and nuclear genes in diploids (2×) and tetraploids (4×) of *A. thaliana* (At), *A. lyrata* (Al) and their hybrids (AtxAl).

**Figure S1.** Breeding strategy applied to develop plant material used in the present study. TT—diploid *A. thaliana*; TTTT*—*tetraploid *A. thaliana*; LL—diploid *A. lyrata*; LLLL*—*tetraploid *A. lyrata*; TL and TTLL *A. thaliana ♀* × *A. lyrata* ♂ diploid and tetraploid, respectively. C_1_ and C_4_—first and fourth generation, respectively, after colchicine treatment. F_1_—first generation after hybridization.

**Figure S2.** CFP and Mitotracker® Red CMXRos colocalization in mitochondria of leaves of *A. thaliana* mt‐ck marker line. Representative confocal images of mitochondria of *A. thaliana* mt‐ck line visualized by CFP (a, d) and Mitotracker dye (b, e). Merged images are shown in (c, f). d–f are details of images a–c (indicated by white square in the right part of image c). Note the lack of Mitotracker signal in some parts of the cell (indicated by white arrows).

**Figure S3.** Changes in expression of chloroplast genes involved in cytonuclear complexes after WGD in leaves of the third generation of *A. thaliana* × *A. lyrata* hybrids (F_3_). Each plot represents the relative transcript abundance (*y* axis) of chloroplast‐encoded genes involved in cytonuclear complexes in diploid (light green) and corresponding polyploid (dark green) plants based on qRT‐PCR in F_3_ *A. thaliana* × *A. lyrata*. Error bars represent the standard error of the mean of the biological replicates. *P < 0.05 (based on two‐tailed Student's *t*‐test).

**Figure S4.** Changes in expression of genes not involved in cytonuclear complexes after WGD in leaves of *A. thaliana* C_4_ and *A. thaliana* × *A. lyrata* hybrids. Each plot represents the relative transcript abundance (*y* axis) of (a) nuclear‐encoded, chloroplast (left) and mitochondria (right) targeted genes that are not involved in cytonuclear complexes in diploid (light green, pink) and corresponding polyploid (dark green, purple) *A. thaliana* C_4_ plants, (b) chloroplast (left) and mitochondria (right) encoded genes that are not involved in cytonuclear complexes in diploid and corresponding polyploid *A. thaliana* × *A. lyrata* plants based on qRT‐PCR. Error bars represent the standard error of the mean of the biological replicates. **P < 0.01, *P < 0.05 (based on two‐tailed Student's *t*‐test).

**Figure S5.** Changes in expression of cytonuclear genes involved in chloroplast and mitochondrial complexes after WGD in leaves of *A. thaliana, A. lyrata*, and *A. thaliana* × *A. lyrata* hybrids using direct normalization of organelle‐encoded Ct values to nuclear‐partner Ct values for chloroplast and mitochondrial cytonuclear genes. Each plot represents a comparison between the log2 values of −ΔCq (ΔCq = Ct organelle − Ct nuclear) (*y* axis), post‐WGD, estimating organelle:nuclear transcript stoichiometry across ploidies. (a) Chloroplast‐encoded, *A. thaliana* (b) Chloroplast‐encoded, *A. lyrata* (c) Chloroplast‐encoded *A. thaliana C4* (d) Chloroplast‐encoded *A. thaliana* × *A. lyrata* (e) Mitochondria encoded, *A. thaliana* (f) Mitochondria‐encoded, *A. lyrata* (g) itochondria encoded *A. thaliana C4* (H) Mitochondria encoded *A. thaliana* × *A. lyrata*. Across all datasets, organelle:nuclear ratios were maintained or increased in tetraploid relative to diploid plants. Error bars denote means and 95% Confidence Intervals. A two‐sided *t*‐test comparing tetraploid versus diploid plants' transcript ratios was performed.

**Table S1.** Primers. The list of oligonucleotides used in the present study.

## Data Availability

The data supporting the findings of this study are available in the supplementary material of this article. All raw data were deposited to Zenodo (https://doi.org/10.5281/zenodo.16901958). Link: https://urldefense.com/v3/__https://doi.org/10.5281/zenodo.16901958__;!!N11eV2iwtfs!pcfe2wsJxSR1TYgeePzfkd00Sp9WpLyd8A1VC0olNVObbIF3lTcDumpFxdu4tXB1qbTYTbdQzt2xWg8jX1DB$.
