# Supplementary figures and images for: Restoring cytonuclear harmony: Distinct strategies in Arabidopsis auto‐ and allopolyploids
Source: Plant J. 2025 Aug 28;123(4):e70451. doi: 10.1111/tpj.70451 (PMC12392245; doi:10.1111/tpj.70451)

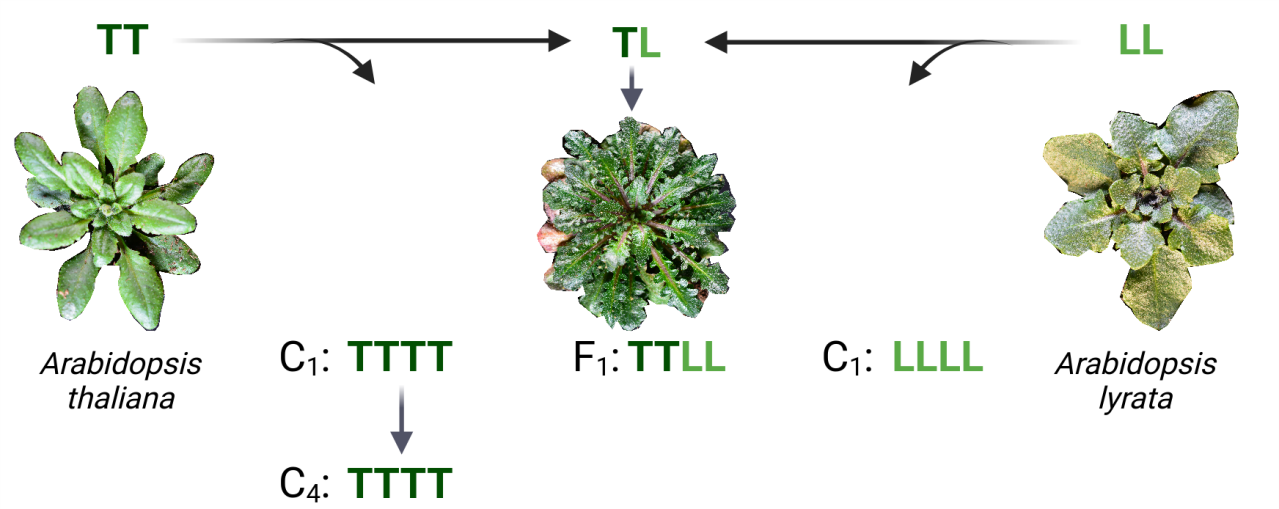

Supplement: Supplementary file 5 — Figure S1. Breeding strategy applied to develop plant material used in the present study. TT—diploid A. thaliana; TTTT—tetraploid A. thaliana; LL—diploid A. lyrata; LLLL—tetraploid A. lyrata; TL and TTLL A. thaliana ♀ × A. lyrata ♂ diploid and tetraploid, respectively. C1 and C4—first and fourth generation, respectively, after colchicine treatment. F1—first generation after hybridization. [file TPJ-123-0-s002.png]

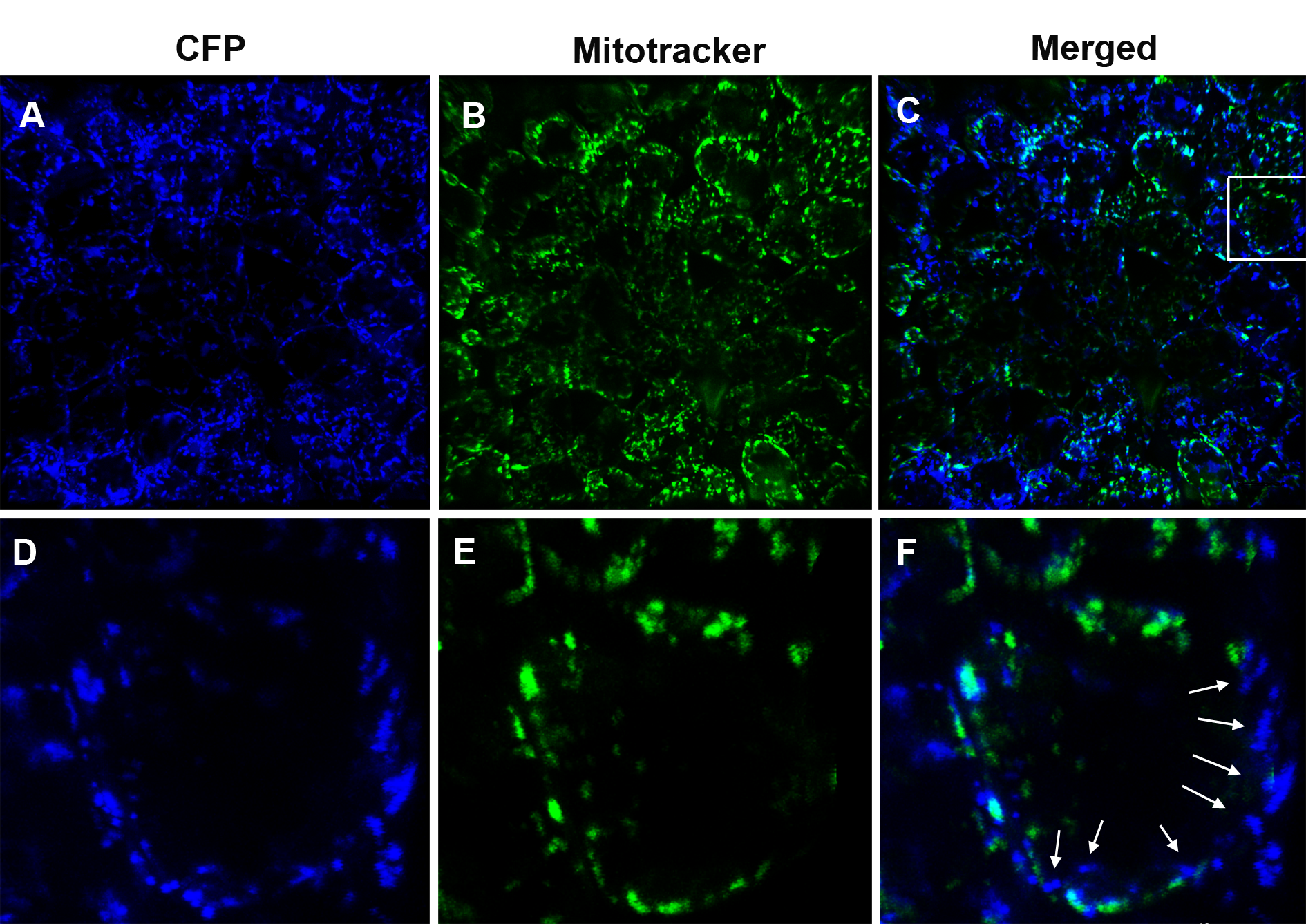

Supplement: Supplementary file 6 — Figure S2. CFP and Mitotracker® Red CMXRos colocalization in mitochondria of leaves of A. thaliana mt‐ck marker line. Representative confocal images of mitochondria of A. thaliana mt‐ck line visualized by CFP (a, d) and Mitotracker dye (b, e). Merged images are shown in (c, f). d–f are details of images a–c (indicated by white square in the right part of image c). Note the lack of Mitotracker signal in some parts of the cell (indicated by white arrows). [file TPJ-123-0-s003.png]

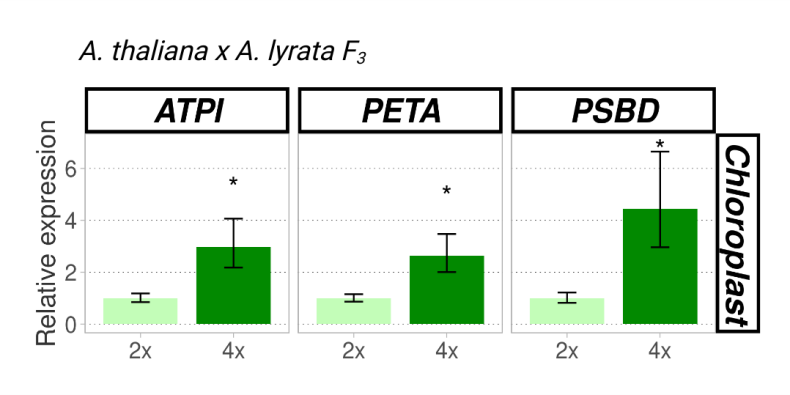

Supplement: Supplementary file 7 — Figure S3. Changes in expression of chloroplast genes involved in cytonuclear complexes after WGD in leaves of the third generation of A. thaliana × A. lyrata hybrids (F3). Each plot represents the relative transcript abundance (y axis) of chloroplast‐encoded genes involved in cytonuclear complexes in diploid (light green) and corresponding polyploid (dark green) plants based on qRT‐PCR in F3 A. thaliana × A. lyrata. Error bars represent the standard error of the mean of the biological replicates. *P < 0.05 (based on two‐tailed Student's t‐test). [file TPJ-123-0-s008.png]

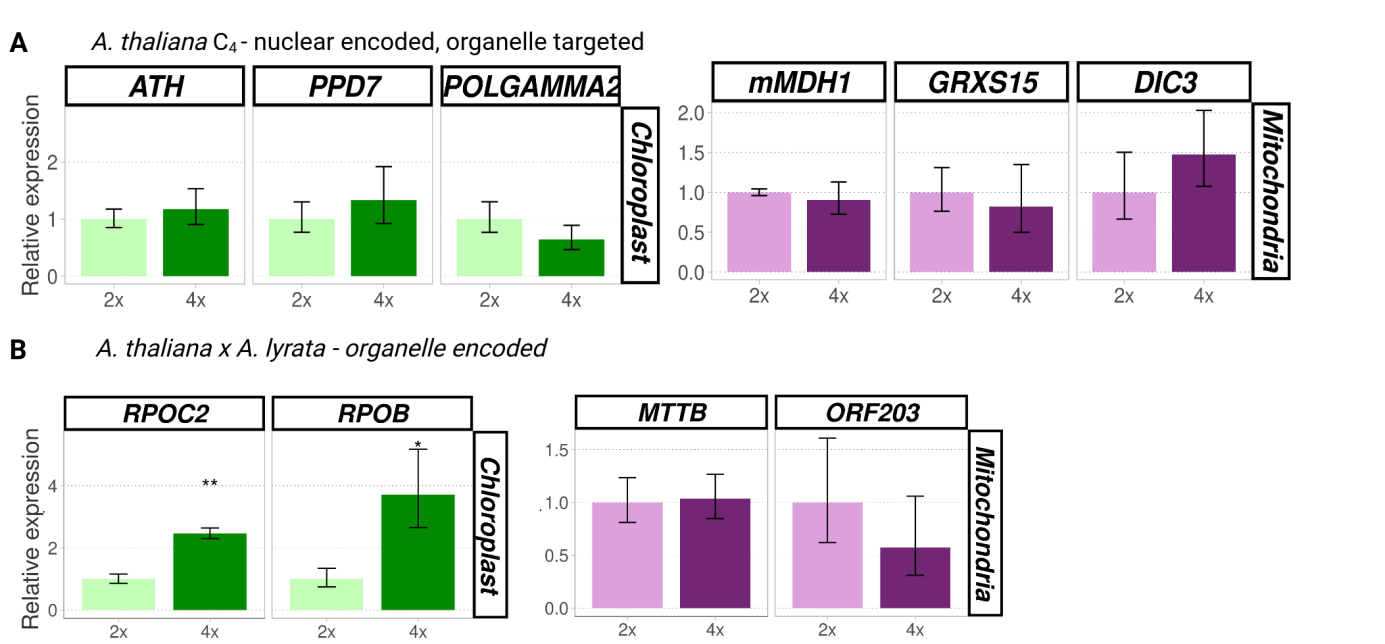

Supplement: Supplementary file 8 — Figure S4. Changes in expression of genes not involved in cytonuclear complexes after WGD in leaves of A. thaliana C4 and A. thaliana × A. lyrata hybrids. Each plot represents the relative transcript abundance (y axis) of (a) nuclear‐encoded, chloroplast (left) and mitochondria (right) targeted genes that are not involved in cytonuclear complexes in diploid (light green, pink) and corresponding polyploid (dark green, purple) A. thaliana C4 plants, (b) chloroplast (left) and mitochondria (right) encoded genes that are not involved in cytonuclear complexes in diploid and corresponding polyploid A. thaliana × A. lyrata plants based on qRT‐PCR. Error bars represent the standard error of the mean of the biological replicates. **P < 0.01, *P < 0.05 (based on two‐tailed Student's t‐test). [file TPJ-123-0-s001.png]

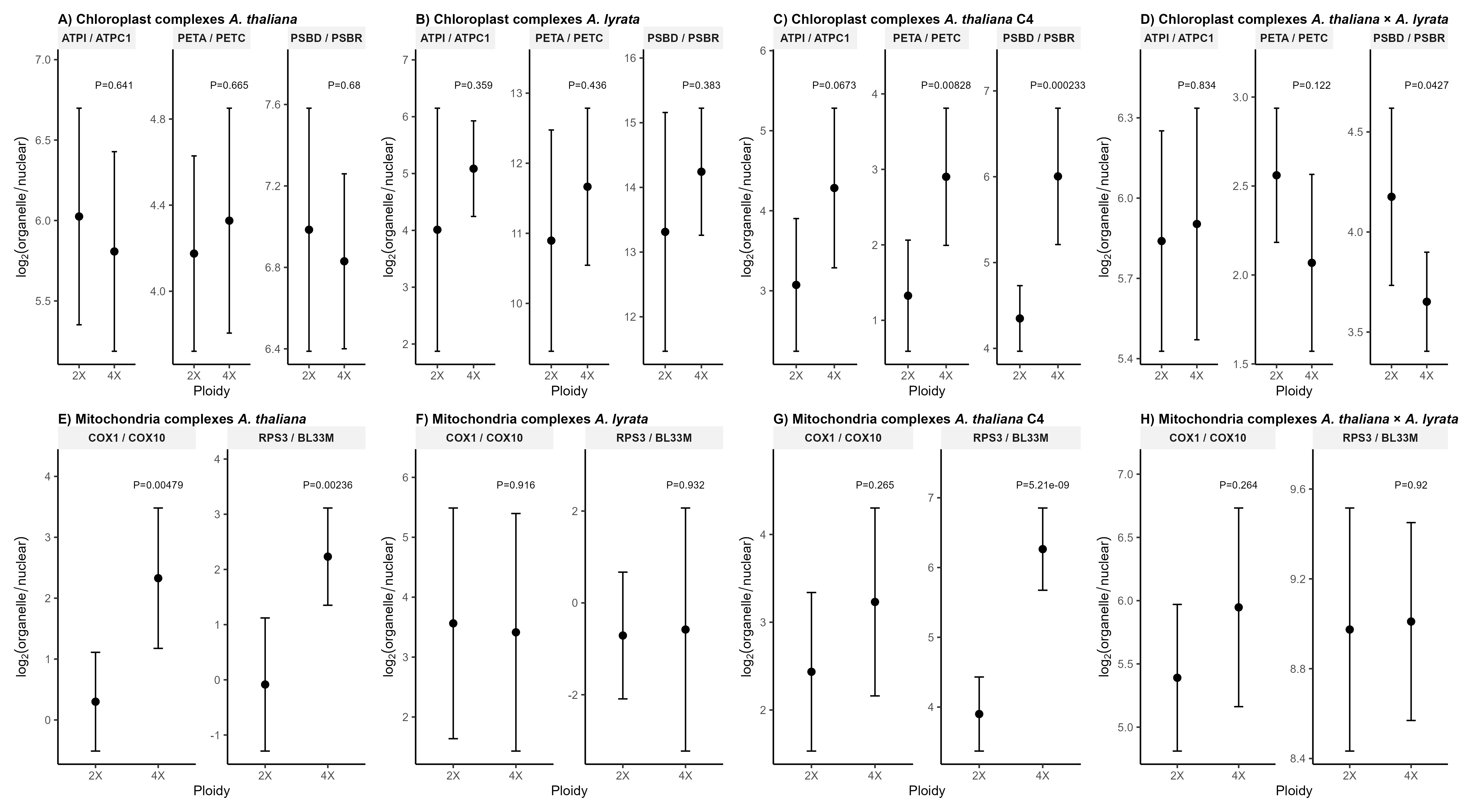

Supplement: Supplementary file 9 — Figure S5. Changes in expression of cytonuclear genes involved in chloroplast and mitochondrial complexes after WGD in leaves of A. thaliana, A. lyrata, and A. thaliana × A. lyrata hybrids using direct normalization of organelle‐encoded Ct values to nuclear‐partner Ct values for chloroplast and mitochondrial cytonuclear genes. Each plot represents a comparison between the log2 values of −ΔCq (ΔCq = Ct organelle − Ct nuclear) (y axis), post‐WGD, estimating organelle:nuclear transcript stoichiometry across ploidies. (a) Chloroplast‐encoded, A. thaliana (b) Chloroplast‐encoded, A. lyrata (c) Chloroplast‐encoded A. thaliana C4 (d) Chloroplast‐encoded A. thaliana × A. lyrata (e) Mitochondria encoded, A. thaliana (f) Mitochondria‐encoded, A. lyrata (g) itochondria encoded A. thaliana C4 (H) Mitochondria encoded A. thaliana × A. lyrata. Across all datasets, organelle:nuclear ratios were maintained or increased in tetraploid relative to diploid plants. Error bars denote means and 95% Confidence Intervals. A two‐sided t‐test comparing tetraploid versus diploid plants' transcript ratios was performed. [file TPJ-123-0-s005.png]
