# Supplementary material for: Restoring cytonuclear harmony: Distinct strategies in Arabidopsis auto‐ and allopolyploids
Source: Plant J. 2025 Aug 28;123(4):e70451. doi: 10.1111/tpj.70451 (PMC12392245; doi:10.1111/tpj.70451)
Supplement: Supplementary file 10 — Table S1. Primers. The list of oligonucleotides used in the present study. [file TPJ-123-0-s010.docx]

**Supporting Files, Tables**

**Table S1. Primers.** The list of oligonucleotides used in the present study.

| Experiment | Gene name-species | Forward Primer | Reverse Primer |
| --- | --- | --- | --- |
| ddPCR | ***NDHB*-AT** | 5’-CAGAGACTACGAGTGTAATAGGA-3’ | 5’-TTCTCAATCTTTTCGACCTTGGTTC-3’ |
|  | ***NDHB*-AL** | 5’-TTCAGAGACTACGAGTGTAATAGGA-3’ | 5’-TTCTCAATCTTTTCGACCTTGGTTC-3’ |
| ddPCR  +  qPCR | ***PSBR*-AT** | 5’-CCCTCGTATTTGAAGCCACA-3’ | 5’-ATTTTGAAGGAGGGACGAGC-3’ |
|  | ***PSBR*-AL** | 5’-TTGTGAGAAAATAATGGCTGCTTCA-3’ | 5’-ACGTATTTGTAAACACCGTATCCCT-3’ |
|  | ***ATPI*-AT** | 5’-TTGGCAAATAGGGGGTTTCC-3’ | 5’-TTTTGGCCGTCAGTTGGAAT-3’ |
|  | ***ATPI*-AL** | 5’-GCTCCTGACCAGTTAGAAACAAAAA-3’ | 5’-TATTAGGTTCCGCAGTTCTAACGAT-3’ |
|  | ***PETA*-AT** | 5’-GGTCCTGTTCCTGGTCAAAA-3’ | 5’-TCCCCTGTTTCCACCTACAT-3’ |
|  | ***PETA*-AL** | 5’-CGGACCCTGCTACTAATAAAGATGT-3’ | 5’-TTTCGTATCCCCCTTTTTCTTTTCG-3’ |
|  | ***PSBD*-AT** | 5’-TTCAATGGTCACCGCTAACC-3’ | 5’-TAGGCACGTAGGTTCAAAGC-3’ |
|  | ***PSBD*-AL** | 5’-ATTTGCACCATCACCATCTTCAAAT-3’ | 5’-TTGCTGTTTTTGTTTCTGTCTTCCT-3’ |
|  | ***ATP6*-AT-AL** | 5’-TCGTGCTGAACCTGGTAAAG-3’ | 5’-GTGACCAAGATGCAAGGGAA-3’ |
|  | ***ATPb1*-AT-AL** | 5’-CTCTGAAGTGTCTGCTTTACTC-3’ | 5’-CGTCCAAGTGAGCAAAAGTTGT-3’ |
|  | ***COX1*-AT-AL** | 5’-TCCTTGGGCTCTTGAACTGA-3’ | 5’-CGTCTCCTTGATAGCTGGAAG-3’ |
|  | ***RPS3*-AT-AL** | 5’-GGTGCTCCTTTCTCAAACCA-3’ | 5’-ATCGTTCGGTTCGGATAAGTC-3’ |
| qPCR | ***ATPC1*-AT-AL** | 5’-TGGTCAAATCAGAACCCGTG-3’ | 5’-GAACAGGGTCTTGCTCGAAT-3’ |
|  | ***PETC*-AT-AL** | 5’-AGAATTTCTTGTCAAGCGTCGAG-3’ | 5’-TGAGTCTTAAGCCATTCCGC-3’ |
|  | ***COX10*-AT-AL** | 5’-GTCTCGACGGAATCAACTGC-3’ | 5’-AGCATTTCCCGTACCCAGAA-3’ |
|  | ***BL33M*-AT-AL** | 5’-ATGTTCATCCGTCTTGTCTCA-3’ | 5’-CGTACTTACGGAACTCGAGC-3’ |
|  | ***RPOB*-AT-AL** | 5’-AATCCTCCTATGCTCCGGAA-3’ | 5’-CCACCCTTTTTCTGAACCCA-3’ |
|  | ***RPOC2*-AT-AL** | 5’-AAATATCCCGACACAGTGGC-3’ | 5’-TGGTTGGTGTAATCCGTTGG-3’ |
|  | ***MTTB*-AT-AL** | 5’-CGGAGGCCTTTTCGACATTT-3’ | 5’-TTGATGTTGCACCCACGAAG-3’ |
|  | ***ORF203*-AT-AL** | 5’-GCGTGTCGTTCGTAATGGAA-3’ | 5’-CCCGAGGCATAAAAGAAGCA-3’ |
|  | ***ATH*-AT** | 5’-CAGGAAAGGAAGACACGGAG-3’ | 5’-ACCAGCTTCTTCCATTGCAA-3’ |
|  | ***POLGAMMA2*-AT** | 5’-TTCCTCCTTTGTCTGTGTGC-3’ | 5’-TTCTGGTTTTCATGCCGACA-3’ |
|  | ***PPD7*-AT** | 5’-GTGACCTGAAAACTACCCCG-3’ | 5’-AAATTCGCACCAATCGCAAG-3’ |
|  | ***DIC3*-AT** | 5’-GAATCCCATCGACGTTGTGA-3’ | 5’-CTTCCTCTGCCACCATCTTC-3’ |
|  | ***GRXS15*-AT** | 5’-AAGTCCCTCCCGATTCTACG-3’ | 5’-GAGCTAAACCCACACTGAGG-3’ |
|  | ***mMDH1*-AT** | 5’-TGGTCATGCTGGGGTTACTA-3’ | 5’-CTTTTGCCTCCACGACTTCT-3’ |

AT and AL refer to *A. thaliana* and *A. lyrata*, respectively.

**Supporting Files, Figure Legends**

**Figure S1. Breeding strategy applied to develop plant material used in the present study.** TT – diploid *A. thaliana;* TTTT – tetraploid *A. thaliana*; LL – diploid *A. lyrata;* LLLL – tetraploid *A. lyrata;* TL and TTLL *A. thaliana ♀ x A. lyrata* ♂ diploid and tetraploid respectively. C_1_ and C_4_ – first and fourth generation respectively after colchicine treatment. F_1_ – first generation after hybridization.

**Figure S2: CFP and Mitotracker® Red CMXRos colocalization in mitochondria of leaves of *A. thaliana* mt-ck marker line.**

Representative confocal images of mitochondria of *A. thaliana* mt-ck line visualized by CFP (A, D) and Mitotracker dye (B, E). Merged images are shown in (C, F). D-F are details of images A-C (indicated by white square in the right part of image C). Note the lack of Mitotracker signal in some parts of the cell (indicated by white arrows).

**Figure S3. Changes in expression of chloroplast genes involved in cytonuclear complexes after WGD in leaves of the third generation of *A. thaliana × A. lyrata* hybrids (F_3_).** Each plot represents the relative transcript abundance (y axis) of chloroplast encoded genes involved in cytonuclear complexes in diploid (light green) and corresponding polyploid (dark green) plants based on qRT-PCR in F_3_ *A. thaliana* × *A. lyrata*. Error bars represent the standard error of the mean of the biological replicates. * *p* < 0.05 (Based on two-tailed Student’s t-test).

**Figure S4.** **Changes in expression of genes not involved in cytonuclear complexes after WGD in leaves of *A. thaliana* C_4_ and *A. thaliana × A. lyrata* hybrids.** Each plot represents the relative transcript abundance (y axis) of (a) nuclear encoded, chloroplast (left) and mitochondria (right) targeted genes that are not involved in cytonuclear complexes in diploid (light green, pink) and corresponding polyploid (dark green, purple) *A. thaliana* C_4_ plants, (b) chloroplast (left) and mitochondria (right) encoded genes that are not involved in cytonuclear complexes in diploid and corresponding polyploid *A. thaliana* × *A. lyrata* plants based on qRT-PCR. Error bars represent the standard error of the mean of the biological replicates. ** *p* < 0.01, * *p* < 0.05 (Based on two-tailed Student’s t-test).

**Figure S5. Changes in expression of cytonuclear genes involved in chloroplast and mitochondrial complexes after WGD in leaves of *A. thaliana, A. lyrata*, and *A. thaliana × A. lyrata* hybrids using direct normalization of organelle-encoded Ct values to nuclear-partner Ct values for chloroplast and mitochondrial cytonuclear genes.** Each plot represents a comparision between the log2 values of −ΔCq (ΔCq = Ct organelle – Ct nuclear) (y axis), post-WGD, estimating organelle:nuclear transcript stoichiometry across ploidies. (A), Chloroplast encoded, *A. thaliana* (B), Chloroplast encoded, *A. lyrata* (C), Chloroplast encoded *A. thaliana C4* (D), Chloroplast encoded *A. thaliana × A. lyrata* (E), Mitochondria encoded, *A. thaliana* (F), Mitochondria encoded, *A. lyrata* (F), Mitochondria encoded *A. thaliana C4* (H), Mitochondria encoded *A. thaliana × A. lyrata*. Across all datasets, organelle:nuclear ratios were maintained or increased in tetraploid relative to diploid plants. Error bars denote means and 95% Confidence Intervals. A two-sided t-test comparing tetraploid vs. diploid plants‘ transcript ratios was performed.

**Supporting Files, Figures**


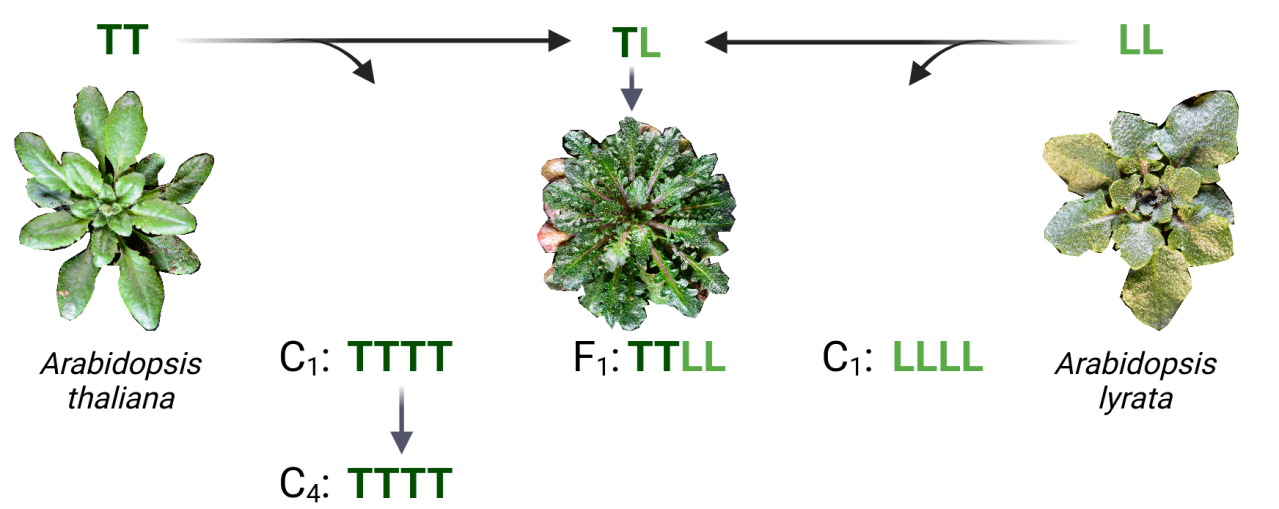


**Figure S1.**

**Figure S2.**


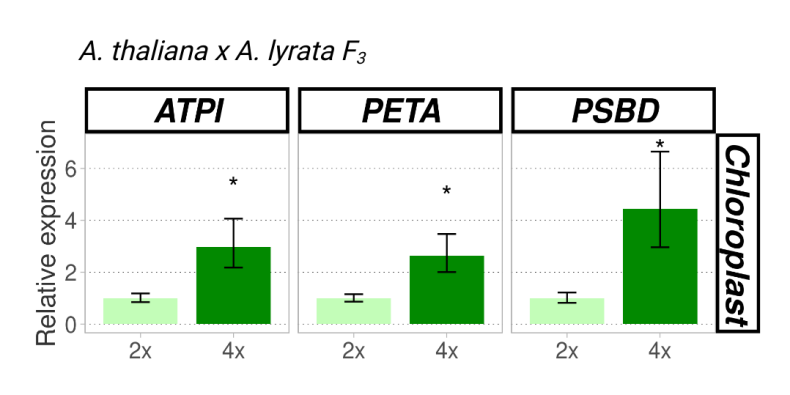


**Figure S3.**


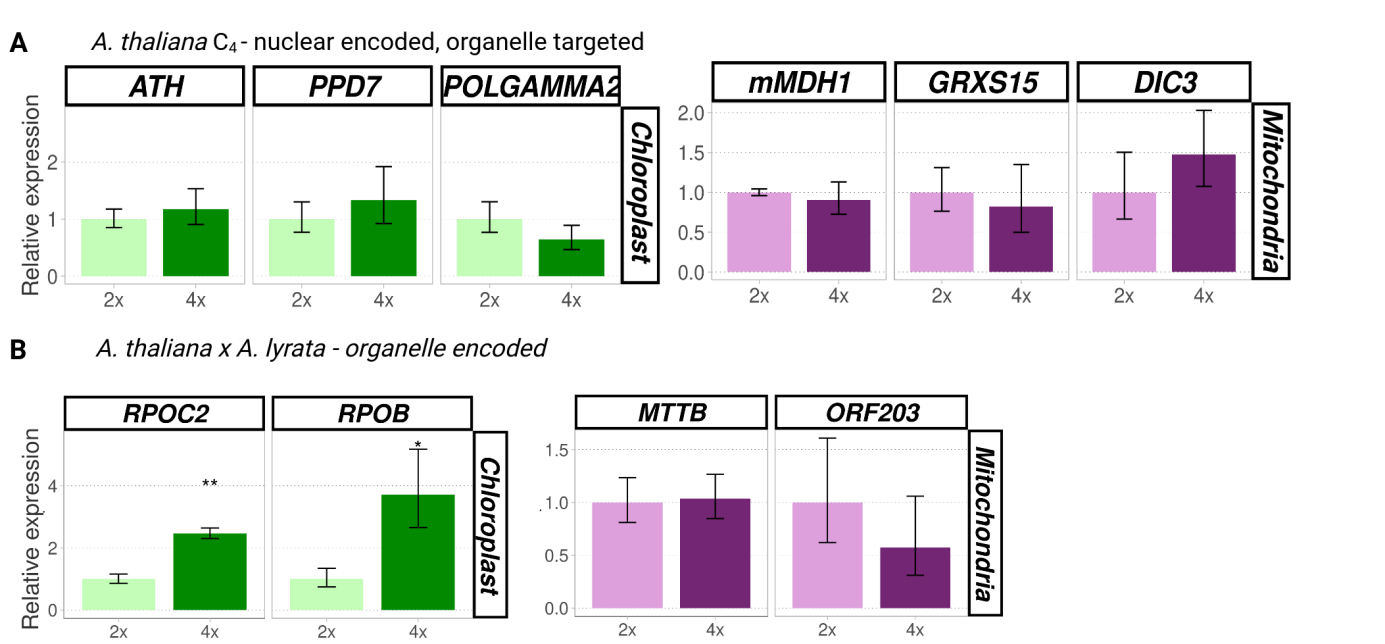


**Figure S4.**

**
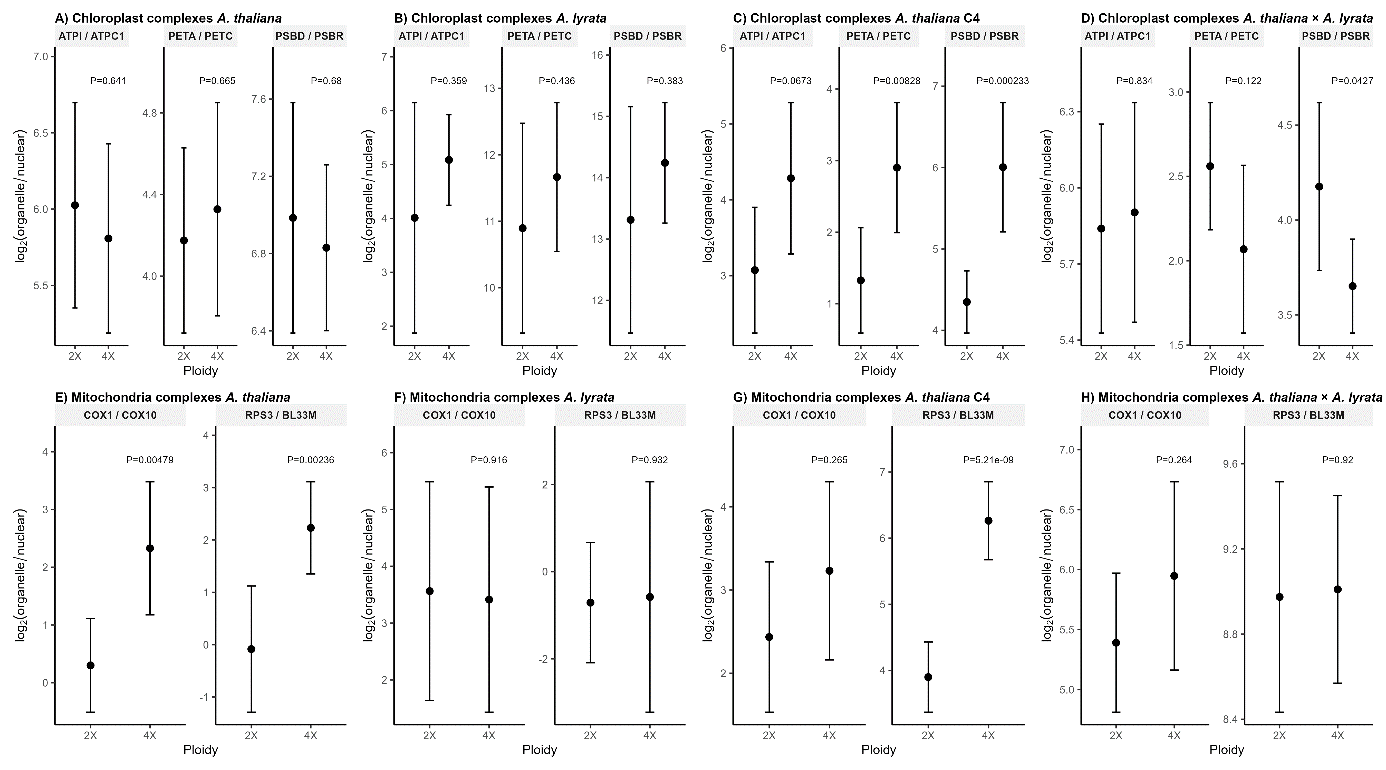
**

**Figure S5.**
